# Laboratory Evolution and Reverse Engineering of *Clostridium thermocellum* for Growth on Glucose and Fructose

**DOI:** 10.1128/AEM.03017-20

**Published:** 2021-04-13

**Authors:** Johannes Yayo, Teun Kuil, Daniel G. Olson, Lee R. Lynd, Evert K. Holwerda, Antonius J. A. van Maris

**Affiliations:** a Department of Industrial Biotechnology, School of Engineering Sciences in Chemistry, Biotechnology and Health, KTH Royal Institute of Technology, Stockholm, Sweden; b Thayer School of Engineering, Dartmouth College, Hanover, New Hampshire, USA; c The Center for Bioenergy Innovation, Oak Ridge National Laboratory, Oak Ridge, Tennessee, USA; University of Buenos Aires

**Keywords:** *Acetivibrio thermocellus*, *Clostridium thermocellum*, *Hungateiclostridium thermocellum*, chemostat cultures, glucose, fructose, laboratory evolution, plate reader screening, ROK protein, reverse metabolic engineering, *cbpA*

## Abstract

The native ability of Clostridium thermocellum to efficiently solubilize cellulose makes it an interesting platform for sustainable biofuel production through consolidated bioprocessing. Together with other improvements, industrial implementation of *C. thermocellum*, as well as fundamental studies into its metabolism, would benefit from improved and reproducible consumption of hexose sugars. To investigate growth of *C. thermocellum* on glucose or fructose, as well as the underlying molecular mechanisms, laboratory evolution was performed in carbon-limited chemostats with increasing concentrations of glucose or fructose and decreasing cellobiose concentrations. Growth on both glucose and fructose was achieved with biomass yields of 0.09 ± 0.00 and 0.18 ± 0.00 g_biomass_ g_substrate_^−1^, respectively, compared to 0.15 ± 0.01 g_biomass_ g_substrate_^−1^ for wild type on cellobiose. Single-colony isolates had no or short lag times on the monosaccharides, while wild type showed 42 ± 4 h on glucose and >80 h on fructose. With good growth on glucose, fructose, and cellobiose, the fructose isolates were chosen for genome sequence-based reverse metabolic engineering. Deletion of a putative transcriptional regulator (Clo1313_1831), which upregulated fructokinase activity, reduced lag time on fructose to 12 h with a growth rate of 0.11 ± 0.01 h^−1^ and resulted in immediate growth on glucose at 0.24 ± 0.01 h^−1^. Additional introduction of a G-to-V mutation at position 148 in *cbpA* resulted in immediate growth on fructose at 0.32 ± 0.03 h^−1^. These insights can guide engineering of strains for fundamental studies into transport and the upper glycolysis, as well as maximizing product yields in industrial settings.

**IMPORTANCE** *C. thermocellum* is an important candidate for sustainable and cost-effective production of bioethanol through consolidated bioprocessing. In addition to unsurpassed cellulose deconstruction, industrial application and fundamental studies would benefit from improvement of glucose and fructose consumption. This study demonstrated that *C. thermocellum* can be evolved for reproducible constitutive growth on glucose or fructose. Subsequent genome sequencing, gene editing, and physiological characterization identified two underlying mutations with a role in (regulation of) transport or metabolism of the hexose sugars. In light of these findings, such mutations have likely (and unknowingly) also occurred in previous studies with *C. thermocellum* using hexose-based media with possible broad regulatory consequences. By targeted modification of these genes, industrial and research strains of *C. thermocellum* can be engineered to (i) reduce glucose accumulation, (ii) study cellodextrin transport systems *in vivo*, (iii) allow experiments at >120 g liter^−1^ soluble substrate concentration, or (iv) reduce costs for labeling studies.

## Abstract (precis)

*C. thermocellum* is an important candidate for sustainable and cost-effective production of bioethanol through consolidated bioprocessing. In addition to unsurpassed cellulose deconstruction, industrial application and fundamental studies would benefit from improvement of glucose and fructose consumption.

## INTRODUCTION

In future low-carbon energy scenarios, sustainable biofuels are foreseen to play a prominent role replacing fossil fuels in ocean shipping, aviation, and long-haul trucking, providing several environmental, societal, and economical benefits (1–4). As a feedstock, lignocellulose is abundant and, as a biofuel, ethanol is currently deployed and needed in near-future transitions to more advanced biofuels (4). In addition, bioethanol has large potential as a sustainable platform chemical for catalytic upgrading to a wide range of products (5). For cost-competitive ethanol production, consolidated bioprocessing, where hydrolysis and fermentation take place in one process unit, would greatly reduce both capital and operating costs compared to alternative enzyme- and yeast-based technologies (6). The anaerobic thermophile Clostridium thermocellum (Ruminiclostridium thermocellum, Hungateiclostridium thermocellum, and Acetivibrio thermocellus [7]) is a promising candidate for consolidated bioprocessing (6, 8, 9). The bacterium outperforms other cellulolytic organisms, as well as industrial cellulases, in efficiently solubilizing lignocellulose (10, 11).

*C. thermocellum* has a growth preference for soluble cellulose hydrolysis products (12, 13). These products, called cellodextrins, consist of β-linked glucose oligomers with degree of polymerization 2–6. For monomeric sugars, such as glucose and fructose, long lag times up to 200 h have been observed (12, 14–17). Indeed, glucose accumulates toward the end of batch cultivations with 10 g liter^−1^ or higher cellulose loadings (18–20). For industrial application, maximizing substrate utilization is crucial for cost-competitive cellulosic ethanol production (6, 21). In contrast to cellobiose, glucose and fructose are cheap and highly soluble model substrates, which in academia can facilitate fundamental studies with *C. thermocellum*, for instance in cellodextrin and cellobiose transport, in labeling studies, in continuous cultivations or at high substrate loadings. Knowledge of the molecular requirements for improved and reproducible growth on monomeric hexoses would therefore be beneficial in both industrial applications of and fundamental studies on *C. thermocellum*.

**FIG 1:**
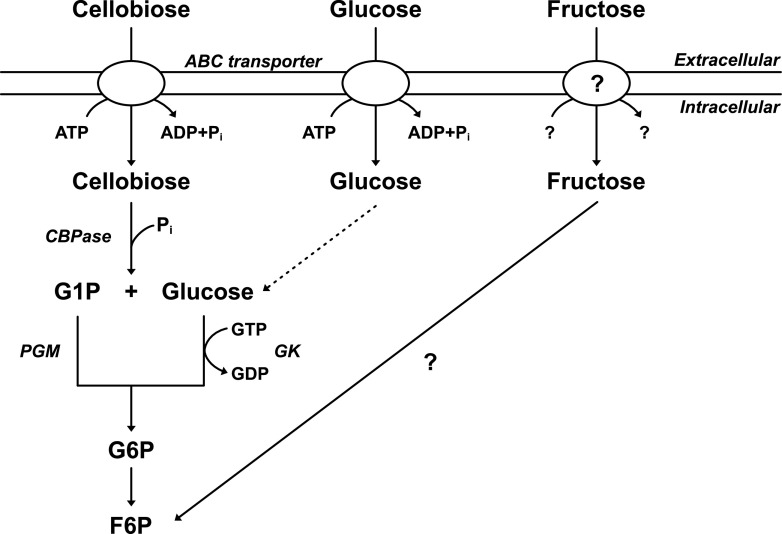
Uptake and upper glycolysis of cellobiose, glucose, and fructose. The question marks represent unknown enzyme or mechanism. Abbreviations: ABC, ATP-binding cassette; CBPase, cellobiose phosphorylase; F6P, fructose 6-phosphate; G1P, glucose 1-phosphate; G6P, glucose 6-phosphate; GK, glucokinase; PGM, phosphoglucomutase.

The metabolism on cellodextrins and glucose has been more studied than fructose metabolism (Fig. 1). Uptake of cellodextrins and glucose has been shown to be ATP dependent (12, 16, 22, 23), which is consistent with *in vitro* affinity studies with several transcribed and translated ATP-binding cassette (ABC)-transport systems (24–26). Intracellularly, cellodextrin phosphorylase acts on a cellodextrin of degree of polymerization *n* (glucose units) and phosphoroclastically cleaves the β-glucosidic bond to form glucose 1-phosphate (G1P) and (*n*-1) cellodextrin. This cleavage continues until cellobiose (*n *=* *2) is reached, which is cleaved to G1P and glucose by a cellobiose phosphorylase (12, 27). The G1P and glucose are converted to glucose 6-phosphate (G6P) by a phosphoglucomutase and a GTP-linked glucokinase, respectively. Thereafter, G6P enters the Embden-Mayerhof-Parnas glycolysis, as described by Zhou et al. (28). Current biochemical knowledge on fructose transport and phosphorylation by *C. thermocellum* is, however, less conclusive. Nochur et al. (23) suggest ATP-linked transport by strain ATCC 27405 and excluded phosphoenolpyruvate-phosphotransferase (PTS) transport. Patni and Alexander (29) suggested PTS-dependent import and phosphorylation to fructose 1-phosphate before conversion by fructose-1-phosphate kinase by strain 651. However, later enzymatic and metabolic studies raised doubts about the purity of strain 651 (28), which additionally is no longer available for further study, necessitating caution in the interpretation of these contrasting result. Independent of the underlying biochemistry, it remains unclear whether the eventual growth on the hexose sugars after the long lag phases requires adaptation or mutation (12, 16, 17, 30).

Laboratory evolution has been successfully used as a tool to maximize substrate utilization, as reviewed elsewhere (31, 32). Beneficial mutations underlying the acquired improved substrate utilization phenotypes can subsequently be identified by whole-genome sequencing and reintroduced into the wild type in order to elucidate the contributing genotype for growth on the nutrient. Such an approach of dissecting a cell, analyzing its components, and subsequently reconstructing the parts that together give a desired phenotype has been named reverse, or inverse, metabolic engineering (33, 34). This can give insights into fundamental biology, such as phenotype-genotype relationships, as well as guide future metabolic engineering to improve the phenotype of the microorganism.

The aim of this study was to (i) reproducibly achieve growth of *C. thermocellum* on hexose sugars through laboratory evolution in chemostats with decreasing concentration of cellobiose and increasing concentrations of glucose or fructose in the medium, (ii) identify possible underlying genetic changes needed for growth on glucose and fructose through whole-genome sequencing of multiple single-colony isolates and reverse metabolic engineering, and through this (iii) elucidate whether mutational events or adaptation are needed for growth of *C. thermocellum* on hexose sugars by analysis of the role of the identified mutations in improving growth and reducing lag time on the monosaccharides.

## RESULTS

### Laboratory evolution for growth on glucose or fructose.

**FIG 2:**
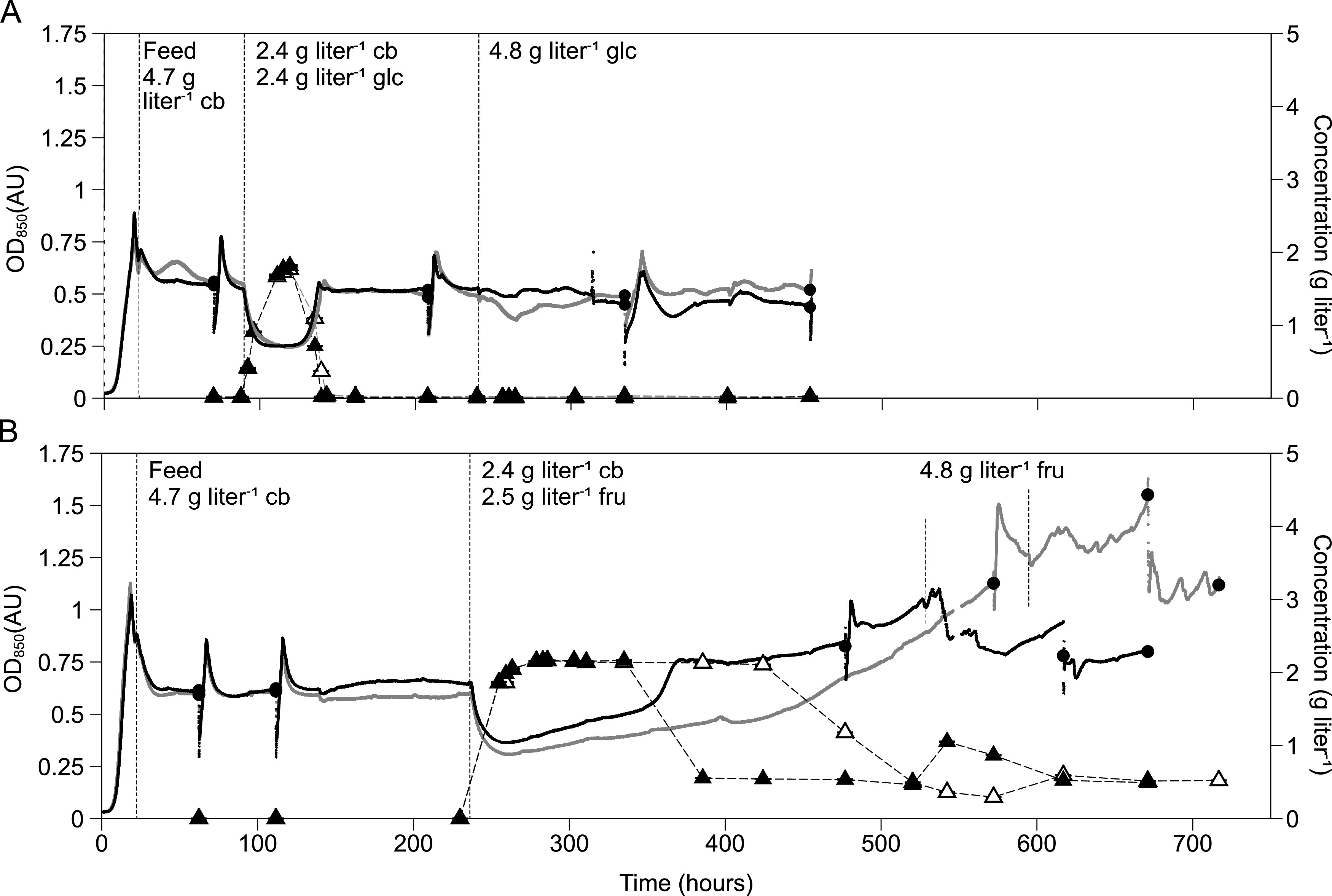
Online optical density (OD_850_; black and gray indicating duplicate cultures) in liquid culture of two parallel carbon-limited chemostats with a dilution rate of 0.10 h^−1^. The cultivation started with a batch phase on 4.7 g liter^−1^ cellobiose and was then continuously fed. The feed vessel was replaced with different combinations of cellobiose (cb), and glucose (glc, A) or fructose (fru, B), as indicated with vertical dashed lines. Samples were withdrawn for measurements of yields and rates at time points indicated with a closed circle. Due to the large sample volumes, the reactor was replenished with fresh medium and consequently led to down- and up-spikes in online OD_850_ directly after each sample point. (A) Switch from cellobiose to glucose. Closed and open triangles represent residual glucose for the respective black and gray replicates. (B) Switch from cellobiose to fructose. Closed and open triangles represent residual fructose for the respective black and gray replicates. Residual substrate concentrations are shown as technical averages ± the standard deviations (*n *=* *3).

To select for *C. thermocellum* growing on glucose or fructose, two sets of duplicate carbon-limited chemostats with decreasing concentration of cellobiose and increasing concentrations of glucose or fructose in the medium were used. Feeding was initiated with 4.7 g liter^−1^ cellobiose, and the first switch was to 2.4 g liter^−1^ cellobiose and either 2.4 g liter^−1^ glucose or 2.5 g liter^−1^ fructose. Thereafter, the feed was switched to 4.8 g liter^−1^ glucose or fructose as the sole carbon and energy source. After the first switch, the biomass concentration decreased approximately 50% (Fig. 2). During this period, the cellobiose fed was utilized while the hexose sugars accumulated. The time point at which the online optical density (OD) increased, indicating improved glucose consumption, was similar for the duplicate chemostats (42 and 43 h, Fig. 2A). In contrast, the onset of fructose uptake took longer (105 and 190 h, Fig. 2B). Biofilm formation was observed in the bioreactors upon switching to fructose-limitation, which made the online OD value estimations unreliable. During this phase, the residual fructose and offline biomass concentration (total organic carbon [TOC]) remained stable (Fig. 2B; see also Fig. S1B in the supplemental material).

Biomass-specific consumption rates and extracellular product yields differed not only between the hexose sugars and cellobiose, but also between the two hexose sugars (Table 1). In order to maintain the same biomass-specific grow rate of 0.10 h^−1^, glucose was taken up 60% faster than cellobiose (1.07 ± 0.03 compared to 0.66 ± 0.06 g g_biomass_^−1^ h^−1^). Consequently, and in line with known metabolism, the biomass yield on glucose was 40% lower (0.09 ± 0.00 g_biomass_ g_substrate_^−1^) than the biomass yield on cellobiose (0.15 ± 0.01 g_biomass_ g_substrate_^−1^). Extracellular product profiles showed a 40% increase in acetate yield on glucose compared to cellobiose and smaller changes in ethanol and formate (*P* < 0.05 based on Student’s *t* test), while the estimated CO_2_ product yield was the same. These changes significantly shifted the acetate/ethanol ratio from 1.2 to 2.0. Surprisingly, fructose was consumed 50% slower than glucose (0.55 ± 0.02 g g_biomass_^−1^ h^−1^) resulting in a high biomass yield (0.18 ± 0.00 g_biomass_ g_substrate_^−1^). The fructose-limited cultures generally showed decreased product yields compared to the other two sugars. Formate was not significantly changed whereas acetate, ethanol, and CO_2_ decreased (24, 32, and 30%, respectively, *P* < 0.05), maintaining the acetate/ethanol ratio constant. The observed residual fructose concentration was 0.5 g liter^−1^, which was significantly higher than the residual 0.02 g liter^−1^ glucose and < 0.01 g liter^−1^ cellobiose concentrations (Table 1), indicating a low affinity for fructose in the evolved cultures.

**TABLE 1:** Physiology of wild-type *C. thermocellum* grown in cellobiose-, glucose-, or fructose-limited chemostats at a dilution rate of 0.10 h^−1^^*a*^

Physiological parameter	Avg ± SD		
	Cellobiose	Glucose	Fructose
Residual substrate concn (g liter^−1^)	<0.01	<0.01	0.51 ± 0.02
*q*_Cellobiose_ (g g_biomass_^−1^ h^−1^)	−0.66 ± 0.06	< 0.01	<0.01
*q*_Glucose_ (g g_biomass_^−1^ h^−1^)	<0.01	−1.07 ± 0.03	<0.01
*q*_Fructose_ (g g_biomass_^−1^ h^−1^)	<0.01	<0.01	−0.55 ± 0.02
Biomass yield (g g_substrate_^−1^)	0.15 ± 0.01	0.09 ± 0.00	0.18 ± 0.00
CO_2_ yield estimate (g g_substrate_^−1^)^*b*^	0.27 ± 0.01	0.28 ± 0.00	0.19 ± 0.01
Ethanol yield (g g_substrate_^−1^)	0.17 ± 0.00	0.14 ± 0.01	0.12 ± 0.00
Acetate yield (g g_substrate_^−1^)	0.20 ± 0.01	0.28 ± 0.01	0.15 ± 0.02
Formate yield (g g_substrate_^−1^)	0.05 ± 0.01	0.06 ± 0.00	0.04 ± 0.00
Lactate yield (g g_substrate_^−1^)	0.01 ± 0.00	0.03 ± 0.01	<0.01
Pyruvate yield (g g_substrate_^−1^)	<0.01	0.02 ± 0.00	0.01 ± 0.00
Malate yield (g g_substrate_^−1^)	<0.01	0.01 ± 0.01	<0.01
Carbon recovery (%)^*c*^	79 ± 1	87 ± 1	67 ± 4

a Data are averages of four biological replicates on cellobiose and two biological replicates on glucose and fructose.

b The CO_2_ yield was calculated as the sum of the following molar yields: ethanol + acetate − formate.

c Carbon recovery was calculated as the ratio between total carbon found in products and total carbon in consumed substrate (see Table S1 in the supplemental material).

### Screening of growth rate and lag time on cellobiose, glucose, and fructose.

To obtain single-colony isolates, a sample from the glucose- or fructose-limited cultures was plated on solid medium with the corresponding monosaccharide. After the first round of plating, colonies were picked, inoculated into liquid medium, and screened for growth characteristics on a plate reader. Based on specific growth rate and lag time, colonies spanning different characteristics were selected (indicated with arrow in Fig. S2 in the supplemental material). These candidates were plated in two subsequent rounds before single-colony isolates were saved as freezer stocks for further analysis. Four single-colony isolates were selected from each chemostat run, resulting in 16 strains in total (Table 2). From here on, the single-colony isolates from the two glucose-limited chemostats are referred to as the glucose isolates (G-strain number) and, similarly, the fructose isolates (F-strain number) represent the group of single-colony isolates from the two fructose-limited chemostats.

**TABLE 2:** Strains used in this study

Strain	Parent strain	Genotype	Accession no.^*a*^	Source or reference
Wild type, or LL1004	DSM 1313	NC_017304.1	DSMZ^*b*^
M1354, or LL345	DSM 1313	DSM 1313 Δ*hpt*	55
AVM009	LL345	DSM 1313 Δ*hpt* Δ*clo1313_0289*	This study
AVM011	LL345	DSM 1313 Δ*hpt* Δ*cbpA*	This study
AVM012	LL345	DSM 1313 Δ*hpt* Δ*clo1313_1857*	This study
AVM013	LL345	DSM 1313 Δ*hpt* Δ*clo1313_1831*	This study
AVM027	AVM013	DSM 1313 Δ*hpt* Δ*clo1313_1831 cbpA*^G148V^	This study
AVM028	AVM011	DSM 1313 Δ*hpt cbpA*^G148V^	This study
AVM055	AVM013	DSM 1313 Δ*hpt* Δ*clo1313_1831* Δ*clo1313_1857*	This study
AVM058	AVM009	DSM 1313 Δ*hpt* Δ*clo1313_0289* Δ*clo1313_1831*	This study
G-LL1516	DSM 1313 evolved in glucose-limited chemostat replicate 1, colony 1	SRX5249377	This study
G-LL1517	DSM 1313 evolved in glucose-limited chemostat replicate 1, colony 2	SRX5249777	This study
G-LL1518	DSM 1313 evolved in glucose-limited chemostat replicate 1, colony 3	SRX5249776	This study
G-LL1523	DSM 1313 evolved in glucose-limited chemostat replicate 1, colony 4	SRX4664279	This study
G-LL1519	DSM 1313 evolved in glucose-limited chemostat replicate 2, colony 1	SRX5249775	This study
G-LL1520	DSM 1313 evolved in glucose-limited chemostat replicate 2, colony 2	SRX5249778	This study
G-LL1521	DSM 1313 evolved in glucose-limited chemostat replicate 2, colony 3	SRX4038392	This study
G-LL1522	DSM 1313 evolved in glucose-limited chemostat replicate 2, colony 4	SRX5249182	This study
F-LL1538	DSM 1313 evolved in fructose-limited chemostat replicate 1, colony 1	SRX4038403	This study
F-LL1539	DSM 1313 evolved in fructose-limited chemostat replicate 1, colony 2	SRX4038408	This study
F-LL1540	DSM 1313 evolved in fructose-limited chemostat replicate 1, colony 3	SRX4038409	This study
F-LL1545	DSM 1313 evolved in fructose-limited chemostat replicate 1, colony 4	SRX4038455	This study
F-LL1541	DSM 1313 evolved in fructose-limited chemostat replicate 2, colony 1	SRX4038419	This study
F-LL1542	DSM 1313 evolved in fructose-limited chemostat replicate 2, colony 2	SRX4038453	This study
F-LL1543	DSM 1313 evolved in fructose-limited chemostat replicate 2, colony 3	SRX4038420	This study
F-LL1544	DSM 1313 evolved in fructose-limited chemostat replicate 2, colony 4	SRX4038454	This study

a Accession numbers refer to raw whole-genome sequence data deposited at the NCBI Sequence Read Archive (https://www.ncbi.nlm.nih.gov/sra). For DSM 1313, this refers to the genomic nucleotide reference sequence at the NCBI (https://www.ncbi.nlm.nih.gov/nuccore/).

b DSMZ, German Collection of Microorganisms and Cell Cultures GmbH, Braunschweig, Germany (www.dsmz.de).

**FIG 3:**
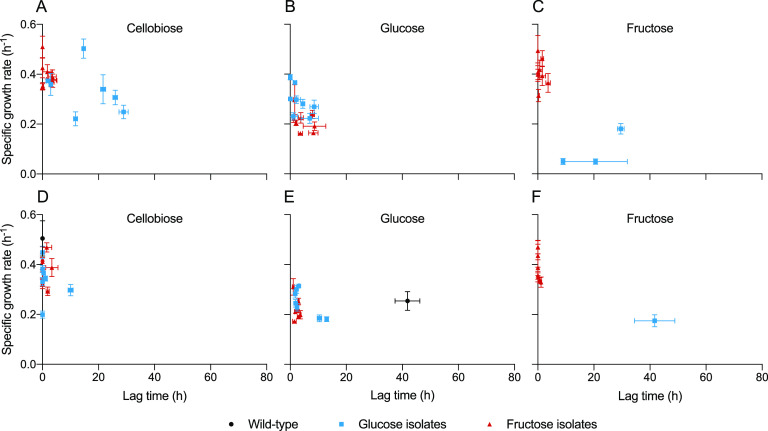
Specific growth rate and lag time on 5 g liter^−1^ cellobiose, glucose, and fructose of single-colony isolates from glucose-limited (blue squares) or fructose-limited (red triangles) chemostats. The isolates were grown in batch cultivations for up to 80 h in a microplate incubated in a plate reader. Inocula for the isolates in panels A to C were grown on the hexose in which they were isolated, whereas in panels D to F the inocula were grown on cellobiose. Wild type (black circle) was used as a control. The data are shown as averages ± the standard deviations for 20 biological replicates for the wild type and three to five biological replicates for the remaining strains.

The specific growth rate and lag time of each single-colony isolate on each of the three sugars were subsequently quantified in microplate cultivations in a plate reader. Prior to a transfer to each of the three sugars, the inoculum was grown on the monosaccharide that the strain was isolated on, or on cellobiose in the case of wild type. As a reference, the wild-type strain grew on cellobiose with 0.50 ± 0.07 h^−1^ (Fig. 3D; see also Table S3 in the supplemental material), whereas on glucose the growth of wild type only commenced after 42 ± 4 h at a lower growth rate of 0.25 ± 0.04 h^−1^ (*P* < 0.01, Fig. 3E). No growth on fructose was observed for the wild type within the time frame of the experiment (80 h). To assess the impact of the laboratory evolution, both hexose isolates were investigated for growth on cellobiose. The fructose isolates as a group had a slightly lower specific growth rate on cellobiose (0.40 ± 0.05 h^−1^, *n *=* *8, *P* < 0.01) than the wild type and demonstrated only minimal lag times (<3.6 h) (Fig. 3A). Among these isolates, F-LL1538 was an outlier with 0.51 ± 0.04 h^−1^ and no lag time on cellobiose (see Table S4). In comparison to the fructose strains, the glucose isolates grew more variably on cellobiose (Fig. 3A). For instance, one strain (G-LL1521) did not grow on cellobiose within 80 h, while another strain (G-LL1519) grew similarly to the wild type at 0.50 ± 0.04 h^−1^ (see Table S4). As a group, the glucose strains (excluding G-LL1521) showed an average specific growth rate on cellobiose of 0.34 ± 0.09 h^−1^ and an average lag time of 15 ± 11 h.

Next, growth and lag time of the hexose isolates on the sugar they evolved on, as well as the alternate hexose sugar, was investigated. On glucose, the glucose isolates as a group grew with 0.29 ± 0.06 h^−1^ (Fig. 3B). On fructose, the fructose isolates as a group grew with 0.41 ± 0.06 h^−1^ (Fig. 3C). Surprisingly, the fructose isolates also demonstrated rapid growth on glucose at 0.21 ± 0.04 h^−1^ with a lag time of 4.7 ± 3.1 h, which was similar to that of the glucose isolates (Fig. 3B). This shows that both groups of isolates grew on glucose with significantly reduced lag times and growth rates comparable to those of the wild type. Interestingly, only one of the glucose isolates (G-LL1520) grew reproducibly on fructose after a 30 ± 1 h lag time at 0.18 ± 0.02 h^−1^. Two of the glucose isolates (G-LL1517 and G-LL1519) showed growth on fructose for only three out of the five experimental replicates at a low growth rate of 0.05 ± 0.01 h^−1^ (see Table S4). The remaining glucose isolates did not grow on fructose within the 80 h of the experiment (Fig. 3C).

### Constitutive growth on glucose or fructose from isolates.

To investigate whether the isolates had adapted or evolved for growth on glucose and fructose, another plate-reader experiment was conducted where the isolates were grown for two transfers in media with 5 g liter^−1^ cellobiose. At midexponential phase, the cultures were used to inoculate a 96-well microplate with 5 g liter^−1^ glucose or fructose. The fructose isolates showed similar strongly reduced lag times for growth on glucose and fructose independent on whether the inocula carbon source was cellobiose or fructose (Fig. 3E and F). Also, the glucose isolates showed a reduced lag time for growth on glucose upon transfer from cellobiose (Fig. 3E). Even though three glucose isolates—G-LL1518, G-LL1522, and G-LL1523—had a slightly increased lag time on glucose, from 1.7 ± 0.6, 0, and 0 h when the inocula were grown on glucose to 13 ± 0.4, 10 ± 1, and 2.3 ± 0.1 h (*P* < 0.01), respectively, these lag times are still more than four times shorter than those for the wild type on glucose (42 ± 4 h) (see Table S3). These observations suggested that the isolates acquired mutations which allowed constitutive growth with no or short lag time on glucose and fructose upon transfer from cellobiose.

### Whole-genome sequencing of isolates revealed targets for reverse engineering.

The 16 single-colony isolates were whole-genome sequenced in order to identify mutations contributing to constitutive growth on glucose and fructose. The sequencing reads were mapped against the DSM 1313 reference genome to find single-nucleotide polymorphisms, insertions, and deletions. All identified mutations are listed in Data Set S1 in the supplemental material. Mutations found in the parent strain (LL1004) were not considered for reverse engineering. A mutation was also excluded for further study if it was found in (i) a hypothetical protein, (ii) a noncoding region far from coding regions, or (iii) genes unlikely contributing to growth on glucose and fructose based on the current genome annotation and knowledge of *C. thermocellum* metabolism.

The fructose and glucose isolates shared mutations in three genes (Table 3). First, the gene *clo1313_1828* (*cbpA* [24]) was frequently mutated in both groups. The gene *cbpA* encodes a periplasmic sugar-binding protein belonging to an ATP-binding cassette (ABC) transport system, which is highly translated (20, 26), and has binding affinity for cellotriose (24). Seven of eight glucose isolates had a single-point mutation g.2140693C>G (the number refers to the position in the genome) that resulted in the amino acid change A173P. The eighth isolate (G-LL1520) had a double mutation g.2140395G>A and g.2140503C>G, which resulted in the amino acid changes A272V and G236A, respectively. All fructose isolates shared a mutation in a different position in *cbpA*, g.2140767C>A, resulting in the different amino acid change G148V.

**TABLE 3:** Identified mutations from whole-genome sequencing of single-colony isolates selected in duplicate glucose- or fructose-limited chemostats^*a*^

Annotation	Locus description	Start region in genome^*b*^	Description^*c*^	Type^*d*^	Glucose-limited replicate 1				Glucose-limited replicate 2				Fructose-limited replicate 1				Fructose-limited replicate 2			
					G-LL1516	G-LL1517	G-LL1518	G-LL1523	G-LL1519	G-LL1520	G-LL1521	G-LL1522	F-LL1538	F-LL1539	F-LL1540	F-LL1545	F-LL1541	F-LL1542	F-LL1543	F-LL1544
*clo1313_0289*^*e*^	Putative transcriptional regulator, CopG family	315368	T → -, Leu15fs	Del	1	1	1
106 bp upstream of *pta*	Phosphate acetyl/butaryl transferase	1407629	- →A	In	1	1	1	1	1	1
*clo1313_1194* *cbpB*^*e*^	Carbohydrate-binding protein	1418197	T → A, Leu8*	SNV	1
		1418737	G → A, Trp188*	SNV	1
		1419366	A → -, Lys398fs	Del	1
		1419411	G → T, Glu413*	SNV	1
*clo1313_1828 cbpA*	Carbohydrate-binding protein	2140395	G → A, Ala272Val	SNV	1
		2140503	C → G, Gly236Ala	SNV	1
		2140693	C → G, Ala173Pro	SNV	1	1	1	1	1	1	1
		2140767	C → A, Gly148Val	SNV	1	1	1	1	1	1	1	1
137 bp downstream of *clo1313_1831*	ROK family protein	2144041	ISCth1	In	0.9	0.9	0.9	0.9	0.9
*clo1313_1831*^*e*^	ROK family protein	2144301	- → T, Ala361fs	In	1	1	1	1	1	1
		2144421	- → T, Ala321fs	In	1
		2144605	A → C, Tyr259*	SNV	1	1
		2145039	C → A, Glu115*	SNV	1
*clo1313_1857*^*e*^	Helix-turn-helix domain protein	2171867	A → T, Trp184Arg	SNV	1	1	1
		2172004	- → A, Tyr138fs	In	1
		2172072	IS120	In	1
		2172368	A → -, Ser17fs	Del	1	1	1	1

a Mutations are reported as the frequency in the sequenced strain.

b Start region in the genome of DSM 1313 with NCBI reference sequence NC_017304.1 (https://www.ncbi.nlm.nih.gov/nuccore/).

c Presented as nucleotide change and, if the mutation was in a coding region, the resulting change in the protein sequence. These protein changes are indicated as nonsynonymous (amino acid change), frameshift (fs), truncated protein (*), or synonymous mutation (-). The number refers to a position in the protein.

d SNV, single-nucleotide variation; Del, deletion; In, insertion.

e Selected for reverse engineering.

Second, the gene *clo1313_1831* was mutated in both groups. Clo1313_1831 is annotated as a member of the Repressor-ORF-Kinase (ROK) family of transcriptional regulators and kinases (35). It has both a helix-turn-helix motif common for repressors and an ATP binding motif common for kinases (36). For seven fructose isolates and three glucose isolates, this mutation resulted in a frameshift or a premature stop codon, likely inactivating the gene product. For the other five glucose isolates (G-LL1516 to G-LL1519, as well as G-LL1523), an insertion sequence ISCth1 (see Fig. S3A) was inserted 137 bp downstream of *clo1313_1831*. The ISCth1 belongs to the IS982 family and contains a transposase within the IS1 protein family (37). While *clo1313_1831* is located on the reverse strand, ISCth1 was on the forward strand.

Third, *clo1313_1857* was mutated in the two sets of fructose isolates and in one glucose isolate. In three fructose isolates (F-LL1542 to F-LL1544) from the same chemostat, the mutation resulted in an amino acid change. The fourth isolate from that chemostat had a transposon insert, IS120 (see Fig. S3B), likely resulting in an inactivated gene. Fructose isolates from the other chemostat all had mutations resulting in an early frameshift in this gene. Also, one glucose isolate contained a frameshift in this gene. According to the genome annotation (36), it encodes a helix-turn-helix domain part of the XRE family of transcriptional regulators (protein ID ADU74910).

The remaining mutations were not shared by the two groups (Table 3). Half the glucose isolates had a frameshift or premature stop codon in *cbpB* (*clo1313_1194*), which is another highly translated sugar-binding protein belonging to an ABC-transport system (20, 24, 26, 36). Another gene, *clo1313_0289*, was frameshifted in three fructose isolates from the same chemostat (F-LL1542 to F-LL1544), likely inactivating the protein. This gene is annotated as a putative transcriptional regulator within the CopG family (36). Since it was mutated in F-LL1542, which lacked a mutation in the *clo1313_1831* ROK gene, it may have had a contributing role to growth on fructose.

### Reverse engineering identified important genes for growth on glucose and fructose.

Fructose isolates not only grew effectively on fructose but also grew similarly to the wild type on cellobiose and similarly to the glucose isolates on glucose. In view of this and the time-consuming nature of gene editing in *C. thermocellum* (38), only mutations found in the fructose isolates were selected for reverse engineering. Based on the whole-genome sequence analysis in the previous section, the following four mutations were selected to investigate their contributions to growth on fructose and glucose through reverse metabolic engineering: knockout of the genes *clo1313_0289*, *clo1313_1831*, and *clo1313_1857* and introduction of the point mutation g.2140767C>A (G148V) into *cbpA*.

**FIG 4:**
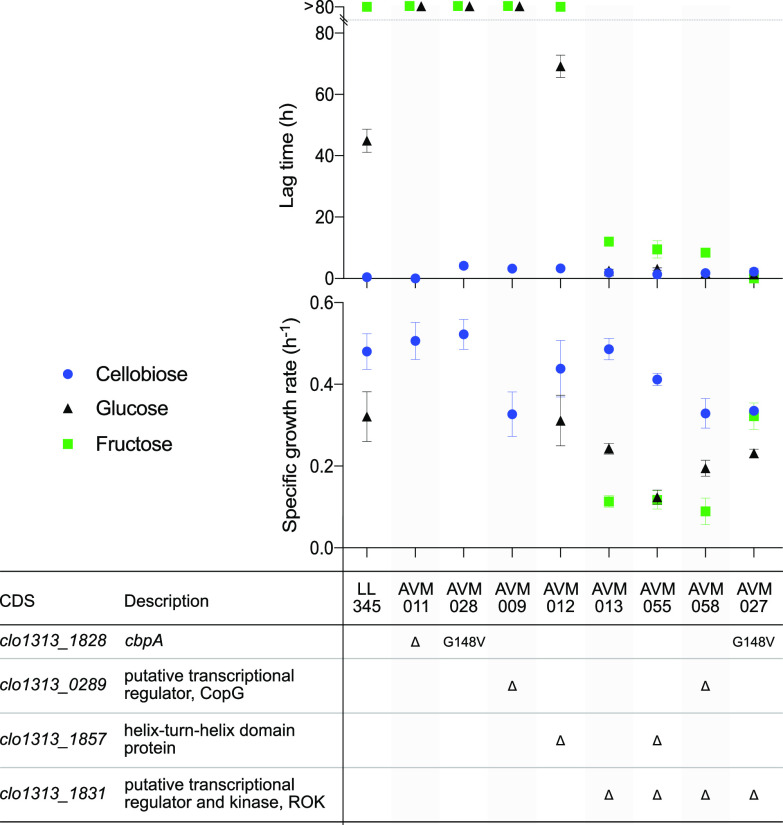
Maximum specific growth rate and lag time on cellobiose (blue circle), glucose (black triangle), and fructose (green square) of reverse engineered strains in batch cultures from a plate reader experiment run for 80 h. The data are shown as averages ± the standard deviations for 23 biological replicates for LL345 and three to five biological replicates for the engineered strains.

These mutations were introduced in the genetically tractable reference strain LL345 (Δ*hpt*). Growth on cellobiose, glucose, or fructose, was characterized in a plate-reader experiment. This reference strain grew readily on cellobiose (0.48 ± 0.05 h^−1^) and slower on glucose (0.32 ± 0.07 h^−1^, *P* < 0.01, Fig. 4). The lag time on glucose was 45 ± 4 h, and the strain did not grow on fructose within the time frame of this experiment (80 h). All this was similar to the previously used wild-type strain.

Introduction of three of the four individual mutations had no beneficial effects and was actually detrimental for growth on at least one of the sugars (Fig. 4). Knockout of *clo1313_1857* (AVM012) resulted in a 53% longer lag time on glucose (69 ± 4 h). The Δ*cbpA* (AVM011), *cbpA*^G148V^ (AVM028) and Δ*clo1313_0289* (AVM009) mutants did not show growth on glucose within 80 h. In addition, Δ*clo1313_0289* (AVM009) grew 32% slower on cellobiose compared to LL345 (0.33 ± 0.05 h^−1^, *P* < 0.01). In contrast, knockout of *clo1313_1831* (AVM013) maintained the high growth rate on cellobiose (0.49 ± 0.03 h^−1^) and enabled growth on fructose, with a specific growth rate of 0.11 ± 0.01 h^−1^ and a lag time of 12 ± 1 h. The lag time on glucose was also significantly reduced to 2.6 ± 0.5 h while maintaining slightly lower growth rate compared to LL345 (0.24 ± 0.01 h^−1^, *P* < 0.01). To test a hypothesis that phosphorylation is limiting growth on fructose, it was investigated whether deletion of the ROK protein Clo1313_1831 resulted in derepression of a fructokinase. Fructokinase activity was not detected in the parent strain LL345, neither ATP- nor GTP-linked (Table 4). On the contrary, the Δ*clo1313_1831* mutant (AVM013) grown on cellobiose showed 0.29 ± 0.02 U mg_protein_^−1^ fructokinase activity with both ATP and GTP. The GTP-linked glucokinase activity did not show a similarly large increase (Table 4).

**TABLE 4:** Glucokinase and fructokinase activity in cell-free extracts from LL345 (Δ *htp*) and AVM013 (Δ *htp* *clo1313_1831*) grown on different carbon sources

Strain	Carbon source	Cofactor	Avg activity ± SD^*a*^	
			Fructokinase activity	Glucokinase activity
LL345	Cellobiose	ATP	<0.05	<0.05
GTP	<0.05	0.25 ± 0.02
AVM013	Cellobiose	ATP	0.29 ± 0.01	<0.05
GTP	0.29 ± 0.02	0.35 ± 0.02
AVM013	Glucose	ATP	0.72 ± 0.04	<0.05
GTP	0.72 ± 0.04	0.12 ± 0.02
AVM013	Fructose	ATP	0.81 ± 0.09	<0.05
GTP	0.76 ± 0.08	0.33 ± 0.03

a Activities are reported as units (U) per mg of total protein in the cell-free extract, where one unit (U) is equal to converting 1 μmol of substrate in 1 min. Data are shown as averages ± standard deviation (*n* = 4).

In comparison to the fructose isolates, growth of Δ*clo1313_1831* (AVM013) showed a longer lag time and lower growth rate on fructose. To investigate the contribution of additional mutations to this phenotype, the *clo1313_1831* deletion was combined with the other three reverse-engineering candidates. Combinations of Δ*clo1313_1831* with Δ*clo1313_1857* and Δ*clo1313_0289*, respectively, yielded the same growth rate and lag time on fructose compared to the parent strain (Δ*clo1313_1831*) (Fig. 4). However, introduction of the single-point mutation *cbpA*^G148V^ in the Δ*clo1313_1831* mutant (AVM027) more than doubled the growth rate to 0.32 ± 0.03 h^−1^ on fructose and abolished the lag time. On glucose, there was no significant changes compared to the single knockout, while the growth rate on cellobiose was reduced by 30% to 0.34 ± 0.01 h^−1^ (*P* < 0.01).

## DISCUSSION

In this study, *C. thermocellum* was successfully evolved for constitutive growth on fructose and glucose. Single-colony isolates from glucose- or fructose-limited chemostats showed no or very short lag time on the respective sugar (Fig. 3A to C). In line with a previous study on agar plates (17), this improved phenotype was constitutive and transgenerational (Fig. 3E and F), suggesting that the chemostat cultures had acquired beneficial mutations. Whole-genome sequencing and subsequent reverse metabolic engineering identified beneficial mutations that are sufficient to enable fast and efficient growth on both glucose and fructose. However, in view of the reproducible short lag time of 42 ± 4 h of the wild type on glucose in batch cultures (*n *=* *20; Fig. 3E), which is difficult to reconcile with the stochastic nature of spontaneous mutations, additional contribution of physiological adaptation to growth of the wild type on glucose cannot be excluded. Growth on glucose and fructose was differentiated with respect to other features as well. In batch cultures, the lower maximum growth rate on glucose compared to both fructose and cellobiose (Fig. 3) could be due to a lower maximum uptake rate (12, 23, 30). Furthermore, glucose-limited cultures showed a lower biomass yield of 0.09 g_biomass_ g_substrate_^−1^ compared to 0.15 g_biomass_ g_substrate_^−1^ on cellobiose, which is similar to a previous report on strain LQRI (12), and reflects the bioenergetic benefit from growing on the disaccharide, in line with the current understanding of metabolism (8). The glucose-limited cultures also produced more acetate, which is stoichiometrically coupled to ATP-formation. Increased acetate production may be to compensate for the lower ATP-yield from glycolysis. In contrast, the biomass yield on fructose was 0.18 g_biomass_ g_substrate_^−1^, similar to a previous report of 0.17 g_biomass_ g_substrate_^−1^ with ATCC 27405 at 0.11 h^−1^ (39), which might reflect different transport and phosphorylation mechanisms compared to glucose.

The genetic change needed in the wild type for constitutive and reproducible growth without a lag time on glucose was inactivation of the gene *clo1313_1831*. On fructose, a point mutation in *cbpA* on top of the *clo1313_1831* deletion was necessary to fully abolish the lag time and achieve a high growth rate (Fig. 4). Inactivation of Clo1313_1831 was likely also necessary in the chemostat evolution, where it was mutated in most isolates. In the few isolates that had no mutation in *clo1313_1831*, its transcription may have been affected by a transposable element inserted downstream of the gene. Transcriptional interference can occur when a strong transposase promoter is inserted “face-to-face” with another weak promoter, in so-called convergent transcription (40, 41). However, further studies into gene expression of those isolates would be needed to confirm such interference. Interestingly, in a previous study, this gene was secondarily mutated in an evolved and engineered strain of *C. thermocellum* (LL1210), which compared to the wild type no longer accumulated glucose (20). Although the contributing role of this mutation was not studied, it may play an important role in light of these results. Deletion of Clo1313_1831, which is a member of the Repressor-ORF-Kinase (ROK) family of transcriptional regulators and kinases, resulted in upregulation of fructokinase activity (Table 4). However, the impact of deleting this putative regulatory protein is likely broader, including for instance transport-related processes, as illustrated by the impact of this deletion for growth on glucose without affecting glucokinase activity. Investigation of the targets of the putative transcriptional regulator Clo1313_1831 by transcriptomics would be of relevance for follow-up studies. The point mutation in *cbpA*, which is suggested to be part of an ABC-transporter system (24) or its signaling cascade, may have increased the affinity of this protein for fructose. Such a modification has been shown *in vitro* in the arabinose-binding protein encoded by *araF* in Escherichia coli, where a single-point mutation in the hinge region increased affinity for galactose 20-fold (42). Transport studies complemented with transcriptomics and/or proteomics in mutant strains could guide further efforts in understanding the molecular basis for the contribution of this *cbpA* mutation to growth on the monosaccharides. Even though the remaining targets for reverse metabolic engineering (*clo1313_1857* and *clo1313_0289*) did not positively contribute to growth on glucose or fructose in batch, these targets may have contributed to improved fitness (e.g., better affinity) in the continuous cultures. In general, regulation of carbon utilization is poorly understood in *C. thermocellum* and these findings, together with further expression studies that for instance might reveal activation of otherwise silent genes (43), might help increase the understanding of how the organism regulates its metabolism on a broader range of carbon substrates.

The present study demonstrated the genetic changes necessary for constitutive and reproducible growth on glucose and fructose without a lag time and can guide engineering of strains for industrial applications and fundamental studies. In industry, high product yields are of key importance for production of low-value high-volume products such as ethanol (21). Studies have shown that *C. thermocellum* accumulates glucose in batch cultivations with industrially relevant cellulose concentrations (18, 19). Also engineered strains for ethanol production show this phenotype (20, 44). In academia, a strain capable of growing on readily available, inexpensive, and highly soluble model substrates facilitates fundamental studies using, e.g., extensive continuous cultures, isotope-based labeling, or high-substrate loadings. Furthermore, these insights also suggest that interpretation of previous studies on *C. thermocellum* using hexose-based medium might be complicated by the occurrence of spontaneous mutations with possible broader regulatory consequences. Future studies of these mutations should include investigation of the effect of these genetic changes on the cellulolytic capability of *C. thermocellum* and on glucose accumulation in high-substrate batch cultivations. Together, these insights can guide engineering of strains for fundamental studies into transport and upper glycolysis, as well as maximized substrate utilization in industrial applications.

## MATERIALS AND METHODS

### Strains and maintenance.

*C. thermocellum* DSM 1313 was obtained from the DSMZ microorganism collection (www.dsmz.de). All strains used in this study are listed in Table 2. Strains were stocked by addition of 25% (vol/vol) glycerol to overnight cultures grown in CTFUD (described by Olson and Lynd [38]) and stored in 1-ml aliquots in cryovials at −80°C. The strains were prepared in a vinyl anaerobic chamber from Coy Laboratory Products (COY Labs [Grass Lake, MI] or TG Instruments [Helsingborg, Sweden]) with 5% H_2_, 10% CO_2_, and 85% N_2_.

### Media and culture conditions.

The defined growth medium used for the chemostats, bottle/tube cultivations and plate-reader experiments was a low-carbon (LC) medium developed by Holwerda et al. (45) with minor modifications. Per liter, LC medium contained 5 g carbohydrate [d-(+)-cellobiose, d-(+)-glucose, d-(–)-fructose, or Avicel PH105, as indicated], 5 g MOPS, 2 g KH_2_PO_4_, 3 g K_2_HPO_4_, 0.1 g Na_2_SO_4_, 0.5 g urea, 0.2 g MgCl_2_·6H_2_O, 0.05 g CaCl_2_·2H_2_O, 0.0035 g FeSO_4_·7H_2_O, 0.025 g FeCl_2_·4H_2_O, 0.1 g l-cysteine HCl monohydrate, vitamins (0.02 g pyrodoxamine dihydrochloride, 0.004 g *p*-aminobenzoic acid, 0.002 g d-biotin, and 0.002 g vitamin B_12_), and trace elements (1.25 mg MnCl_2_·4H_2_O, 0.5 mg ZnCl_2_, 0.125 mg CoCl_2_·6H_2_O, 0.125 mg NiCl_2_·6H_2_O, 0.125 mg CuSO_4_·5H_2_O, 0.125 mg H_3_BO_3_, and 0.125 mg Na_2_MoO_4_·2H_2_O). For continuous cultivations, MOPS was omitted, cysteine was increased to 1 g liter^−1^, and trace element concentrations were increased five times. LC medium for agar plates used in single-colony isolation contained 1 g liter^−1^ cysteine and 0.8% (wt/vol) agar and was prepared in the anaerobic chamber.

The final medium was prepared from sterile stock solutions as described by Holwerda et al. (45): solution A contained carbohydrates at final concentration; solution A* contained 50-fold-concentrated MOPS compared to its concentration in the final medium; solution B contained 25-fold-concentrated KH_2_PO_4_, K_2_HPO_4_, and Na_2_SO_4_; solution C contained 50-fold-concentrated urea; solution D contained 50-fold-concentrated MgCl_2_, CaCl_2_, FeSO_4_, FeCl_2_, and l-cysteine-HCl; solution E contained 50-fold-concentrated vitamins; and solution TE contained 1,000-fold-concentrated trace elements. All solutions were prepared with ultrapure water (MilliQ system from Millipore [Billerica, MA] or Purelab Chorus from AB Ninolab [Stockholm, Sweden]). All stocks were prepared in 125- to 500-ml Wheaton serum bottles (DWK Life Sciences, Millville, NJ), sealed with blue butyl rubber stoppers (Chemglass Life Sciences, Vineland, NJ), and purged extensively by 20 45-s cycles of vacuum and nitrogen gas of ultrahigh purity (N5.0). For bottle and plate cultivations, solutions B and D were autoclaved (at 121°C for 20 min) and the remaining solutions were filter sterilized (0.2 μm PES) into preautoclaved serum bottles and then purged extensively. Suppliers of chemicals and gasses are reported in Table S5 in the supplemental material.

The medium was finalized by adding all stocks to solution A. For small-volume cultivations (≤5 ml), medium was aliquoted into serum tubes (18 × 150 mm; Chemglass Life Sciences). The bottle or tube was then purged with 5 45-s cycles of vacuum and a gas mix of 20% CO_2_ and 80% N_2_ in order to provide CO_2_ to initiate growth.

All cultivations were carried out at 55°C. Serum bottles and tubes were incubated in a Jeio Tech ISS-4075R incubator (Milmedtek AB, Karlskrona, Sweden) at 180 rpm.

### Laboratory evolution in chemostats.

Inocula for the chemostats were prepared by propagating a freezer stock on MTC medium with Avicel (45) and then transferring to LC medium with Avicel. Before all Avicel was consumed, as observed by settled particles, the culture was aliquoted in 5-ml serum bottles and stored at −80°C.

Chemostat cultivations were performed as described by Holwerda et al. (46). Cultivations were carried out in two 0.5-liter round-bottom glass bioreactors (Sartorius Q-plus system; Sartorius Stedim, Bohemia, NY) with a 0.3-liter working volume. The volume was kept constant using an electric level sensor. Agitation was set at 200 rpm with a six-blade Rushton impeller. The pH was measured with a Mettler-Toledo pH probe (Columbus, OH) and maintained at 7.0 by base addition (2 M KOH). A DASGIP OD4 (10-mm path length) biomass monitor from Eppendorf (Hauppauge, NY) recorded the near-infrared (850 nm) OD *in situ*. Off-gas passed through a condenser (4°C) and a water lock to minimize oxygen diffusion into the reactor. Feed medium was prepared in a 10-liter narrow-top reservoir bottle with bottom-hose outlet (Kimble KIMAX from Fisher Scientific, Pittsburgh, PA) and a rubber head-plate with stainless-steel tubes. It was autoclaved (at 121°C for 2.0 to 2.5 h) with water and then continuously purged with N_2_ gas. The medium stock solutions were filter-sterilized into the feed vessel in alphabetic order. The feed vessel was connected to both bioreactors through a precalibrated peristatic pump (Watson-Marlow, Falmouth, Cornwall, England). The weights of both the feed vessel and collected effluent were recorded and used to correct the feed rate to ensure an accurate dilution rate. The headspace of the bioreactor was purged with 20% CO_2_ and 80% N_2_ for 2 to 4 h before inoculation. After inoculation, the culture was grown in a batch phase with no overhead purging until it reached an OD_850_ peak (16 to 24 h). A medium feed of 30 ml h^−1^ was started, corresponding to a dilution rate of 0.10 h^−1^, and the headspace was purged with 5 ml min^−1^ N_2_ gas. At specific time points, the feed vessel was aseptically replaced with various carbohydrate mixtures. The feed medium at the start contained 4.7 g liter^−1^ cellobiose and was switched to a mixture of 2.4 g liter^−1^ cellobiose and either 2.5 g liter^−1^ fructose or 2.4 g liter^−1^ glucose, as indicated. Fructose cultures were switched after 240 h to facilitate collection of additional quantitative data. The final switch was to 4.8 g liter^−1^ fructose or glucose.

Sampling for determining rates and yields was done after at least four residence times on each combination of carbon sources with a smaller than 5% change in biomass concentration during one volume change. After withdrawal of large sample volumes, the reactor volume was brought back to the working volume level with an equal volume of fresh medium. Smaller samples (8 ml) were collected throughout the cultivation.

### Single-colony isolation.

The single-colony isolation consisted of three rounds of plating with one screening step in between rounds. In detail, a continuous-culture sample was transferred to an anaerobic chamber, plated in 10-fold dilution series on solid LC medium with glucose or fructose, and incubated until colonies were visible and distinct. Seven colonies from each bioreactor were picked and screened for growth rate and lag time on the different sugars. The colonies were grown in serum tubes with 4 ml of LC medium with glucose or fructose to an OD_600_ of 0.4 to 0.6 and then used to inoculate a 96-well plate with 200 μl per well of LC medium with either 5 g liter^−1^ cellobiose, glucose, or fructose. The plate was incubated with shaking at 55°C, and the OD_600_ was measured over 72 h in a modified BioTek PowerWave XS plate reader, as described by Olson and Lynd (47). After the screening, selected colonies were plated in 100-fold dilution series in two subsequent rounds to ensure colonies were based on single cells. After the last round, colonies were grown in liquid LC medium with glucose or fructose. At an OD_600_ of 0.6 to 1.0, aliquots were stocked in 5-ml serum bottles and stored at −80°C. The pellet of the remaining culture was sent for whole-genome sequencing. The culture purity was checked by 16S rRNA sequencing, as well as by whole-genome sequencing. No contamination was detected in the single-colony isolates by either method.

### Analytical techniques.

The supernatant from chemostat samples was acidified by adding 35 μl of 10% sulfuric acid (wt/wt) to 700 μl of supernatant. After centrifugation, it was filtered (0.22-μm nylon) and stored at 4°C until analysis. Ethanol, acetate, formate, lactate, pyruvate, malate, glucose, cellobiose, and fructose in the supernatant were quantified by HPLC (Waters, Milford, MA) using an Aminex HPX-87H column (Bio-Rad, Hercules, CA) equipped with a refractive index detector and UV detector. The column was run with 0.5 ml min^−1^ of 2.5 mM H_2_SO_4_ at 60°C. Cell dry weight samples were measured in triplicate by filtering 5-ml samples through preweighed 0.2-μm GTTP Isopore membrane filters (Merck Millipore, Ltd.), washing with an equal volume of ultrapure water, and drying at 100°C for 24 h before weighing. For analysis of pellet total nitrogen (TN) and total organic carbon (TOC), frozen cell pellets were thawed and washed twice with ultrapure water. The cell TN and TOC were analyzed in a Shimadzu TOC-Vcph TOC analyzer with a TN unit and ASI-V autosampler added (Shimadzu Scientific Instruments, Columbia, MD), using an acidified glycine standard as described previously by Holwerda et al. (45). The offline OD was measured at 600 nm in triplicate in a Thermo Scientific Genesys 335901 visible spectrophotometer.

### Plate-reader cultivation and screening.

In order to quantify the growth rate and lag time of isolates and engineered strains, plate-reader cultivations were performed with cellobiose, glucose, or fructose. A freezer stock was propagated in two serial transfers on 5 to 50 ml of LC medium. During exponential growth of the second transfer at an OD_600_ of 0.5 to 1.0, the culture was brought into the anaerobic chamber and used to inoculate a preheated 96-well plate (sterile, clear, flat-bottom, non-surface-treated Eppendorf cell-culture plate with moat from Sigma-Aldrich, Stockholm, Sweden). The plates contained 200 μl of LC medium per well and were sealed with sterile adhesive SealPlate film (Sigma-Aldrich). A BioTek Epoch 2 plate reader from AH Diagnostics AB (Solna, Sweden) incubated the plate at 55°C with continuous double orbital shaking (425 cpm, 3 mm). The plate reader measured the OD at 600 nm every 5 min and was run for 80 h. Each strain was grown in at least five replicates on each carbon source with a distribution covering different parts of the plate. The strains were inoculated to the same starting OD (0.02). On each plate, at least six wells with media were used as negative controls.

A script to analyze the plate-reader OD data was written in Python 2.7 (from the Anaconda Distribution) and was deposited at Github (https://github.com/johannesyayo/plate_reader_analytics). Blanks were averaged at each time point and subtracted from each well. The standard deviation between the blanks was below 0.006. OD values below this threshold were considered as noise. Following this, the specific growth rate was calculated in two steps. In the exponential phase, the specific growth rate is constant and can be calculated as the slope of the natural logarithm of the OD. First, the maximum slope was found for a time frame of 5 h. Second, the exponential phase of the growth curve was expanded with 5-min steps if the new slope was ≥95% of the maximum slope and *R*^2^ was above 0.995. The time until the start of this exponential phase was defined as the lag phase. Manual curation was applied to remove outliers. In case of diauxic growth due to carry-over of a different carbon source from the inoculum, the lag time was manually adjusted as the time between the end of the initial growth phase and the beginning of the main exponential phase.

### Resequencing.

Whole-genome sequencing was used to verify strain construction and check for secondary mutations. Raw data are available from the NCBI Sequence Read Archive (see the accession numbers in Table 2). DNA was submitted to the Joint Genome Institute (JGI) for sequencing with an Illumina MiSeq instrument. Unamplified libraries were generated using a modified version of Illumina’s standard protocol. First, 100 ng of DNA was sheared to 500 bp using a focused ultrasonicator (Covaris). The sheared DNA fragments were size-selected using SPRI beads (Beckman Coulter). The selected fragments were then end repaired, A tailed, and ligated to Illumina compatible adapters (IDT, Inc.) using KAPA Illumina library creation kit (KAPA Biosystems). Libraries were quantified using KAPA Biosystems’ next-generation sequencing library qPCR kit and run on a Roche LightCycler 480 real-time PCR instrument. The quantified libraries were then multiplexed into pools for sequencing. The pools were loaded and sequenced using the appropriate Illumina reagent kit for a 2 × 150-bp indexed run to generate paired-end reads.

Data were analyzed with CLC Genomics Workbench v12 (Qiagen). First, sequences were trimmed for quality (>0.001) and ambiguous residues (maximum of 1). Then, 2,500,000 reads were sampled to give an average read depth of about 105. Reads were mapped to the NC_017304.1 reference genome, using the default parameters, except that the similarity fraction was increased from 0.8 to 0.95 to reduce mapping errors. The preliminary alignment was improved by two passes of local realignment. Mutations were identified using the “basic variant detection” and “structural variant” tools. Single-nucleotide polymorphisms were filtered to eliminate those supported by fewer than 35% of reads or 10 total reads at a given position. Breakpoints were matched to known transposons within the *C. thermocellum* genome by BLAST (48).

### Strain construction.

Plasmids and primers used in this work are shown in Tables 5 and 6, respectively. The backbone plasmid for deletions and insertions was pDGO145 (49), which is derived from pDGO-68 (50) but with a low-copy E. coli origin of replication p15a instead of pUC19. Genomic DNA was extracted using a GeneJet kit from Thermo Fisher Scientific (Stockholm, Sweden). Primers (Invitrogen, Thermo Fisher Scientific) were designed to amplify fragments from genomic DNA with overhangs via PCR using Thermo Scientific Phusion High-Fidelity DNA polymerase. The primers used to amplify *cbpA* for plasmid pJY16 were designed with the specific point mutation g.2140767C>A (G148V). The PCR fragments were assembled with the backbone plasmid using Gibson assembly (51) in an E. coli BL21 derivate (New England Biolabs catalog number C2566I purchased from BioNordika AB, Solna, Sweden). Purification of PCR amplicons and plasmid DNA was done using commercially available kits from GeneJET (Thermo Fisher Scientific). Gene editing in *C. thermocellum* was performed as previously described by Olson and Lynd (38), through transformation by electroporation with a deletion or insertion plasmid, followed by several rounds of positive and negative selection using homologous recombination. For gene insertion or simultaneous gene deletion and insertion, the gene of interest was placed between the 5′ flank and the 3′ flank on the plasmid. For the deletion of *clo1313_0289*, pJY8 was designed with an extra 5′ flank instead of an internal region due to the small gene size. As a first step in the strain construction, the genes *clo1313_1857*, *clo1313_1831*, *clo1313_0289*, and *cbpA* were deleted individually using plasmids pJY3, pJY5, pJY8, and pJY12, respectively, resulting in strains AVM012, AVM013, AVM009, and AVM011 (Table 2). In AVM011, the gene *cbpA* with point-mutation g.2140767C>A (G148V) was inserted using plasmid pJY16, resulting in strain AVM028. Second, Δ*clo1313_1831* was combined with the other gene modifications. The gene *clo1313_1857* was deleted in AVM013 (Δ*clo1313_1831*) using plasmid pJY3, resulting in strain AVM055. The single-point mutation was introduced into AVM013 (Δ*clo1313_1831*) by simultaneous deletion of the native *cbpA* and insertion of *cbpA*^G148V^ using pJY16, resulting in the strain AVM027. The double mutant Δ*clo1313_1831* Δ*clo1313_0289*, named AVM058, was constructed from AVM009 (Δ*clo1313_0289*) using plasmid pJY5. The culture purity (see below) and final genotype was confirmed by Sanger sequencing (Eurofins Genomics, Edersberg, Germany).

**TABLE 5:** Plasmids used in this study

Plasmid	Description	GenBank accession no.	Source or reference
pDGO145	Plasmid backbone for deletion/insertion	KY852359	49
pJY3	Deletion of *clo1313_1857*	MW353731	This study
pJY5	Deletion of *clo1313_1831*	MW353732	This study
pJY8	Deletion of *clo1313_0289*	MW353733	This study
pJY12	Deletion of *cbpA*	MW353734	This study
pJY16	Simultaneous deletion of *cbpA* and insertion of *cbpA*^G148V^	MW353735	This study

**TABLE 6:** Primers used in this study

Primer ID	Sequence (5′ to 3′)^*a*^	Product
0224	GATATCGCCTCGTGATACGC	pDGO145 backbone
0225	CAGCTGCTAATAGTAGTGAAAAAATCAG	pDGO145 backbone
0226	CTGAACTACTGGGCCAGGTATG	p_gapDH_-*cat*-*hpt* selection cassette from pDGO145
0227	ATCGTGGGAATAGGCATGG	p_gapDH_-*cat*-*hpt* selection cassette from pDGO145
0116	cttttcggttagagcggcattatccctgattttttcactactattagcagctgGCTGATTATTTGGGCATTACTC	Internal region of *clo1313_1857* with overhangs matching pDGO145 backbone
0117	acattcacgccctccccatgctttaatacatacctggcccagtagttcagGATATAAGGCAGACTGCTCTCT	Internal region of *clo1313_1857* with overhangs matching p_gapDH_-*cat*-*hpt* casette
0216	aaaaagactctggttttggaaagctTTGTGATATAATTGTAATTCT	5′ flank to *clo1313_1857* with overhangs matching its 3′-flank
0123	cattattatcatgacattaacctataaaaataggcgtatcacgaggcgatatcAATCACACTTTCATCCTG	5′ flank to *clo1313_1857* with overhangs matching pDGO145 backbone
0217	atcaatgcatcctcgggcaaaaaaatcttttccatgcctattcccacgatCTTACGGAGTTAAGCATGTATCC	3′ flank to *clo1313_1857* with overhangs matching p_gapDH_-*cat*-*hpt* casette
0166	taaaagaattacaattatatcacaaAGCTTTCCAAAACCAGAGTC	3′ flank to *clo1313_1857* with overhangs matching its 5′ flank
0171	cttttcggttagagcggcattatccctgattttttcactactattagcagctgGGTTTTTCTTTGTCGGAGTGGATG	Internal region of *clo1313_1831* with overhangs matching pDGO145 backbone
0172	acattcacgccctccccatgctttaatacatacctggcccagtagttcagAGCTATCGCTTCTCCGCTTG	Internal region of *clo1313_1831* with overhangs matching p_gapDH_-*cat*-*hpt* casette
0173	tataatgacggaaagggaggtgctgCTCTTTAGTTAATGCATTTCATTTTTCGC	5′ flank to *clo1313_1831* with overhangs matching its 3′ flank
0174	cattattatcatgacattaacctataaaaataggcgtatcacgaggcgatatcCCTGCATCCTGAGAGTCCTTAG	5′ flank to *clo1313_1831* with overhangs matching pDGO145 backbone
0175	atcaatgcatcctcgggcaaaaaaatcttttccatgcctattcccacgatCTTTGGAAGCCGCCAGAATTG	3′ flank to *clo1313_1831* with overhangs matching p_gapDH_-*cat*-*hpt* casette
0176	aaaatgaaatgcattaactaaagagCAGCACCTCCCTTTCCGTCA	3′ flank to *clo1313_1831* with overhangs matching its 5′ flank
0181	atcaatgcatcctcgggcaaaaaaatcttttccatgcctattcccacgatAAGTAAAACCCCCACTTCTTTTTTGGG	5′ flank of *clo1313_0289* with overhangs matching p_gapDH_-*cat*-*hpt* casette
0182	cattattatcatgacattaacctataaaaataggcgtatcacgaggcgatatcGTGTTGGGACAATAAATTACGAACTGG	5′-flank of *clo1313_0289* with overhangs matching pDGO145 backbone
0183	atatatctccgcgtttaattaccacAAGTAAAACCCCCACTTCTTTTTTGGG	5′ flank to *clo1313_0289* with overhangs matching its 3′ flank
0184	acattcacgccctccccatgctttaatacatacctggcccagtagttcagGTGTTGGGACAATAAATTACGAACTGG	5′ flank to *clo1313_0289* with overhangs matching p_gapDH_-*cat*-*hpt* casette
0185	cttttcggttagagcggcattatccctgattttttcactactattagcagctgGCCGTACTTTTCAATCTCTTCAAGCC	3′ flank to *clo1313_0289* with overhangs matching pDGO145 backbone
0186	caaaaaagaagtgggggttttacttGTGGTAATTAAACGCGGAGATATATTC	3′ flank to *clo1313_0289* with overhangs matching its 5′-flank
0202	cttttcggttagagcggcattatccctgattttttcactactattagcagctgGAGGATGCAGCTAAAGCAATTGG	Internal region of *cbpA* with overhangs matching pDGO145 backbone
0203	acattcacgccctccccatgctttaatacatacctggcccagtagttcagCTTTGATTCTGCAACTGCTGTCG	Internal region of *cbpA* with overhangs matching p_gapDH_-*cat*-*hpt* casette
0204	tatccttaaaaatgtaaggaggtaaGAATAAAATAAAATCAGCATGAAAGGA	5′ flank to *cbpA* with overhangs matching its 3′ flank
0205	cattattatcatgacattaacctataaaaataggcgtatcacgaggcgatatcCATCGTGACTGTCACTTCG	5′ flank to *cbpA* with overhangs matching pDGO145 backbone
0206	atcaatgcatcctcgggcaaaaaaatcttttccatgcctattcccacgatGGAATACCCGTTCTCTTCATC	3′ flank to *cbpA* with overhangs matching p_gapDH_-*cat*-*hpt* casette
0207	ctttcatgctgattttattttattcTTACCTCCTTACATTTTTAAGG	3′ flank to *cbpA* with overhangs matching its 5′-flank
0365	ttcggttagagcggcattatccctgattttttcactactattagcagctgGAATAAAATAAAATCAGCATGAAAGGA	5′ flank to *cbpA* with overhangs matching pDGO145 backbone
0366	acgccctccccatgctttaatacatacctggcccagtagttcagCATCGTGACTGTCACTTCG	5′ flank to *cbpA* with overhangs matching p_gapDH_-*cat*-*hpt* casette
0363	tgatgagtactatccaaagaaataaGAATAAAATAAAATCAGCATGAAAGGA	5′ flank to *cbpA* with overhangs matching the first part of the gene
0364	tattatcatgacattaacctataaaaataggcgtatcacgaggcgatatcCATCGTGACTGTCACTTCG	5′ flank to *cbpA* with overhangs matching pDGO145 backbone
0361	atcaatgcatcctcgggcaaaaaaatcttttccatgcctattcccacgatGGAATACCCGTTCTCTTCATCA	3′ flank to *cbpA* with overhangs matching p_gapDH_-*cat*-*hpt* casette
0362	atactaaaaatcttttcatgaaaaaTTACCTCCTTACATTTTTAAGGA	3′ flank to *cbpA* with overhangs matching the second part of the gene
0371	gaaccggtaactatgcagcag**t**tcagaaggCGGCTGAGTTCCTGGTACCG	*cbpA* first part with overhangs matching second part of the gene. Point mutation in overhang
0358	ctttcatgctgattttattttattcTTATTTCTTTGGATAGTACTCATCAAC	*cbpA* first part with overhangs matching its 5′ flank
0359	tatccttaaaaatgtaaggaggtaaTTTTTCATGAAAAGATTTTTAGTATTATTGC	*cbpA* second part with overhangs matching its 3′ flank
0372	agtttaccaacggtaccaggaactcagccgCCTTCTGA**A**CTGCTGCATAG	*cbpA* second part with overhangs matching the first part of the gene. Point mutation in priming sequence

a Annealing sequences are shown in uppercase letters; overhangs are shown in lowercase letters. The point mutation is underscored and boldfaced.

### Culture purity.

Culture purity was assessed in chemostats, isolates, and genetically modified strains, using 16S rRNA primers from Integrated DNA Technologies (IDT, Coralville, IA) (forward primer, 5′-AGA GTT TGA TCC TGG CTC AG-3′; reverse primer, 5′-ACG GCT ACC TTG TTA CGA CTT-3′). The sequenced PCR product was compared to the *C. thermocellum* DSM 1313 genome. Microscopy was also used during the continuous cultivations. The culture purity was also assessed by analysis of whole-genome sequencing reads. For all strains, >99.94% of reads mapped to the *C. thermocellum* genome. No contamination was detected in this work.

### Enzyme activity assays.

Cell-free extracts were prepared as described previously (49, 50). Cells were grown in 50 ml of LC medium with 5 g liter^−1^ cellobiose, glucose, or fructose. In mid-exponential phase (OD 0.6 to 1.5), they were harvested by centrifugation at 6,500 × *g* for 15 min at 4°C and washed twice with 100 mM Tris-HCl buffer (pH 7.5 at 25°C). Resuspended cell pellets were stored at −20°C until analysis. Upon thawing on ice, cells were centrifuged at 6,500 × *g* for 15 min at 4°C and resuspended in 100 mM Tris-HCl (pH 7.5 at 25°C) with 2 mM dithiothreitol and 10 mM MgCl_2_. Cell extracts were incubated with 60 kU Ready-Lyse lysozyme (Nordic Biolabs, Täby, Sweden) for 50 min at room temperature, followed by incubation with 25 U of DNase I (RNase-free; Thermo Scientific) for 30 min at 37°C. The lysate was centrifuged at 15,000 × *g* for 20 min. The supernatant, i.e., cell-free extract, was stored on ice and used on the same day. The total amount of protein was determined by a Bradford assay with bovine serum albumin as standard (52). Suppliers of chemicals are reported in Table S5 in the supplemental material.

Enzymes were assayed aerobically in a Cary 50 UV-visible spectrophotometer with a single-cell Peltier element from Varian AB (Solna, Sweden). The reduction of NAD(P)^+^ to NAD(P)H was measured at 340 nm in quartz cuvettes (Sigma-Aldrich) with 1-cm path length and 1-ml reaction volume. The temperature was maintained at 55°C using a Peltier element. To convert the change in absorbance to concentration, an extinction coefficient of 6.22 AU mmol^−1^ liter cm^−1^ for NAD(P)H was used. Enzyme activities are expressed as μmol min^−1^ mg_protein_^−1^. To confirm proportionality between the activity and the amount of cell-free extract, two different concentrations of cell-free extract were assayed in duplicate.

Fructokinase was measured by an enzyme-coupled assay similar to that described by Sebastian and Asensio (53). Fructose 6-phosphate formed by fructokinase was converted to G6P by phosphoglucose isomerase, which in turn was converted to 6-phospho-glucono-lactone by NADP^+^-dependent G6P dehydrogenase. The fructokinase assay mixture contained 2 mM fructose, 5 mM MgCl_2_, 2 mM NADP^+^, 2 mM ATP or GTP, 50 mM Tris-HCl (pH 7.5 at 55°C), 2.2 U of G6P dehydrogenase (from Saccharomyces cerevisiae; Sigma-Aldrich G7877), 2 U of G6P isomerase (from S. cerevisiae; Sigma-Aldrich P5381), and 50 or 100 μl of cell-free extract. The reaction was started by the addition of ATP or GTP.

Glucokinase was assayed similarly by coupling the formation of G6P to NADP^+^ reduction via G6P dehydrogenase (28). The assay mixture contained 2 mM glucose, 5 mM MgCl_2_, 60 mM KCl, 2 mM NADP^+^, 2 mM ATP or GTP, 50 mM Tris-HCl (pH 7.5 at 55°C), 2.2 U of G6P dehydrogenase (from S. cerevisiae; Sigma-Aldrich G7877), and 50 or 100 μl of cell-free extract. The reaction was started by the addition of ATP or GTP.

Lactate dehydrogenase was assayed routinely in order to verify the quality of the cell-free extract (see Table S6). The formation of lactate from pyruvate was monitored by measuring the oxidation of NADH (54). The assay mixture contained 10 mM pyruvate, 1 mM fructose 1,6-bisphosphate, 0.22 mM NADH, 200 mM Tris-HCl (pH 7.3 at 55°C), and 50 or 100 μl of cell-free extract. The reaction was started by the addition of pyruvate.

### Data analysis.

Student’s *t* test was used for unpaired comparison between values in this study.

### Data availability.

Measurement data from the chemostats to calculate yields and rates are presented in Table S2. The resulting carbon yields for calculating the carbon balances are presented in Table S1. The whole-genome sequencing data from the single-colony isolates of the evolved strains were deposited to the NCBI Sequence Read Archive (https://www.ncbi.nlm.nih.gov/sra) with accession numbers listed in Table 2. A full list of mutations identified is in Data Set S1 in the supplemental material. Plate-reader data for growth rate and lag time calculations of evolved and engineered strains are available in Data Set S2. The script used to analyze growth parameters based on the plate-reader OD data is available at Github (https://github.com/johannesyayo/plate_reader_analytics).

## Supplementary Material

Supplemental file 1

Supplemental file 2

Supplemental file 3
