# Supplementary material for: Laboratory Evolution and Reverse Engineering of Clostridium thermocellum for Growth on Glucose and Fructose
Source: Appl Environ Microbiol. 2021 Apr 13;87(9):e03017-20. doi: 10.1128/AEM.03017-20 (PMC8091016; doi:10.1128/AEM.03017-20)
Supplement: Supplemental file 3 [file AEM.03017-20-s0003.pdf]

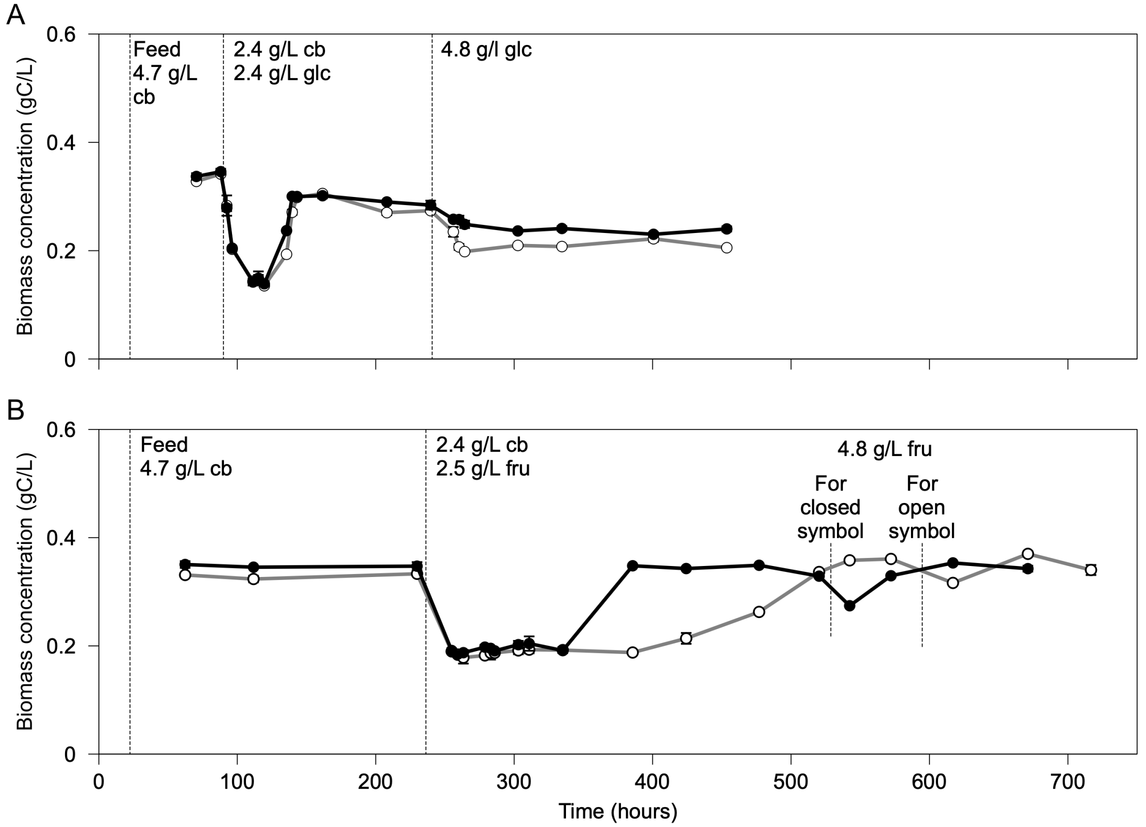

FIG. S1. Total organic carbon (TOC) of cell pellet samples from duplicate carbon-limited chemostats (represented as open and closed symbols) run anaerobically at 55 °C with a dilution rate of 0.1 h^{-1}. The feed vessel was replaced with different combinations of sugars, as indicated with horizontal dashed lines. A: cellobiose (cb) and glucose (glc). B: cellobiose and fructose (fru). Data are shown as technical average ± standard deviation (*n* = 3).

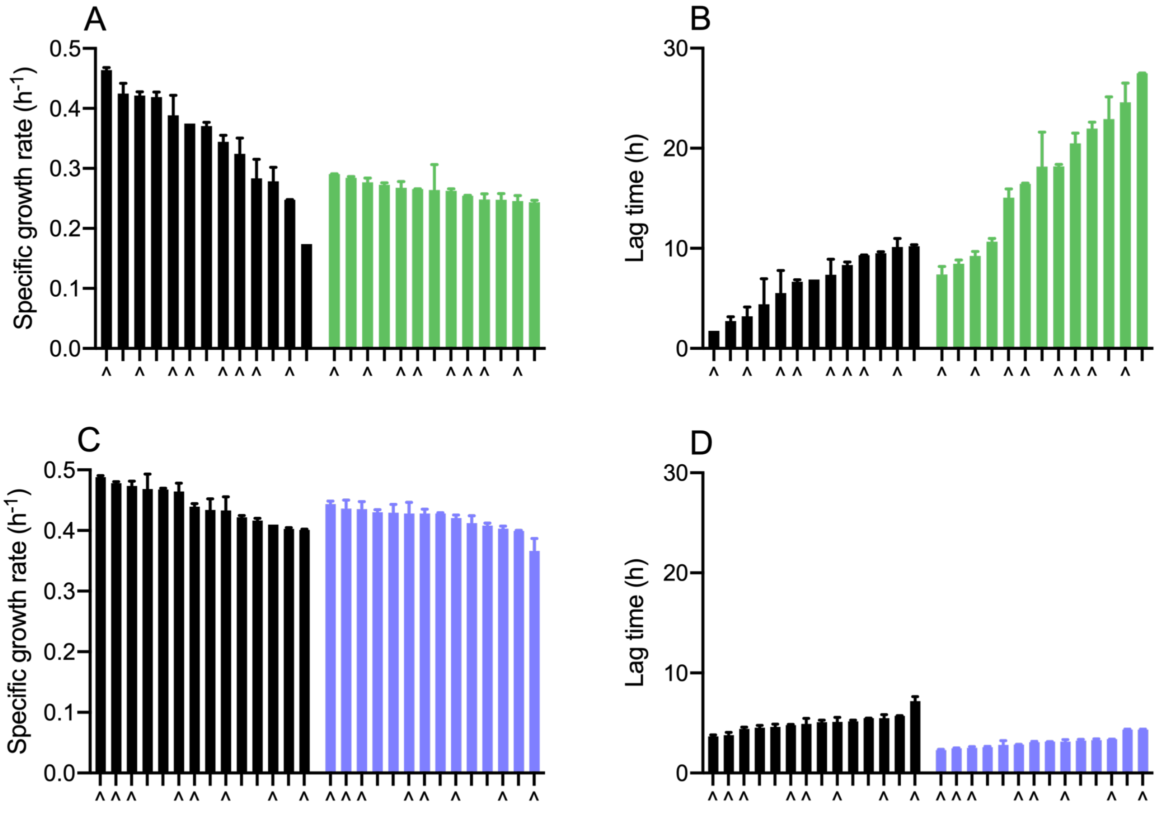

FIG. S2. Screening of growth characteristics during single-colony isolation after one round of plating from carbon-limited chemostats. From the agar plates, several colonies were propagated in liquid cultures with the carbon source they were isolated in. From the liquid cultures, a 96-well plate was inoculated containing cellobiose (black), glucose (green), or fructose (blue), and run in batch in a plate reader. OD_{600} was measured over time for 70 hours. A: specific growth rate on cellobiose and glucose for colonies from duplicate glucose-limited chemostats. B: lag time on cellobiose and glucose for these glucose-isolated colonies. C: specific growth rate on cellobiose and fructose for colonies from duplicate fructose-limited chemostats. D: lag time on cellobiose and fructose for these fructose-isolated colonies. Arrows (^) indicate selected colonies for further isolation. W: wild type. Data are shown as average ± standard deviation (*n* = 2).

A

```
1  TTAATTTAAC TTGTTGTGCT AGCTTTTTTT ACAGGTAAAA ATTGCGAATG AAAAACTCCA ACATAATATT GTAACCTATC ATTAAACACC AAACTAATGA
2  TAGGAGGTCA CAATATATGA TGGAGCTTGA TAAAAATTAT TATATCAAAG AAATTGAAAA CTTAAAAGAC TTTGTAACTG TCGCCTACAT CATAATTGAC
3  GACATTTACC AGAAGGTAAC TCCACCACAC ATTGCAAATC GCTGTAACAT CAATAATTCG GTTATGAGCG ATAGTGAAAT AATTACCATA TGTATTGTTG
4  GAGAGTTACT CACTATCGAC TCTGAGAAAG CTTGGTTGGG TTTCTGTAAG AAGAATATGA GAGACCTGTT TCCCAAATTC TGTGACAGGA CCAGGTTCAA
5  TAGAACCCGC AGAAACCTGC ACGCAGTAAT TGAGGAAATC AGAAAAGAAC TCTCAAACCT TACAGGGTAT GCCCAGCAAC CTTACCGGAT AGTAGATAGT
6  ATTCCTATAC CGGTGTGCAA ATTTGGAAGA GCCAAGTTCC ATAAAACATT CCGTGGTTTC GGAGCAACTT ATGGAAAATG TCCTTCCAAA AAAGAAATAT
7  ATCTGGGCTA TAAGCTGCAT ATGCTTGCAA CCCTGGATGG TTTTATAACT GATTTTGCAA TTACACCTGC CAATGTTGAT GATCGTGCTG GTGTATGGGA
8  CCTGATTGAT TCTTACCGGC GGATAACCTT AATTGGAGAT AAGGGATATA TTGGAACAGA ATTTGCTGTT GAACTAAAAG AGGAAAAGGA AATAGAGATC
9  CTACCTGTTA AAAGGAGCAA CAGCAAGTTA CAGTTTCCGA AAGCTATAAG GCAATTAATC TTCAAATTAA GGCGCCGGAT AGAGACTTCG GCTTCTCAGC
10 TTACCCAGCA ACTCAATATA GAGAAGGTGC TTGCAAAGTC GTACTGGGGA TTTTTAGCCA GGGTAAAAAC CAAGCTTTTA GCCTACAACT TATGTTATTA
11 CATCAATAAG CTTATAGGCC GTGATATTAA TTTTTCGAGG ATCAAAGAGT TAGTATTTGG TTAAAAAATT TAATTCCTCA AACTCAGTAG GAATACTATA
12 GGTGTTTGAG TTGCCATAAT ATTCCTTGAG CCTGTAAACT ATCAGGTAGC ACAACAGATT AAA
```

B

```
1  CAAAATATAA TGATACCAAA AATTAAGACA GACAAAACAG CCCAAATAAG TTAGAATGGA ACTATGGAAA AGAGAAAACA TTTTACACCT GAACAAAAAG
2  CAAAAATAGT GATTGAGGTC ATCAAGGGAG AAAGAACGCT GAATGAGATT GCTGCAGAAT ATGGAATTCA TCCAAACCTG TTAAGTCGCT GGAAGACTGA
3  ATTCATAAGC AATGCGGGCA GAGTATTCAG CAAGGAAACT GATGAAGTAG AGAAGGTCAA ACAGTCGTAT GAAAAGGAGA AGGACGAACT GCTTAAGCAA
4  ATTGGTCAAC TATCATATGA GGTTGCCTGG CTTAAAAAAA AATCTGGCCT CCTCTAAATC CCGAGAAGAC CGCATGAAAA TGATTGATAG AAATGAGAAG
5  AAACTCAGCA TAACAAGGCA AGCAGAATTA TTGAGCTTAA ACCGTACGAG CGTTTACTAC AAGCCTGCTC CGGTAAATGA GGAGGAATAC CTGATTAAGC
6  GTATCATTGA TGAAATTTAC GCGTCTTATC CGGAATATGG CTATCGCAGG ATGACAAGTA TATTGAACAA GGATTATCAC ATTCATATCA ATCGAAAACG
7  GACCCGGCGT TATATGAGGG AAATGGGCAT ACATGGATTC TGTCCTGGCC CCAACCTCAG CAAACGAATA CATGGTAAGA ATTTGTATCC ATATCTGTTG
8  AGAAACTTGA AAATTGATCA TCCTAATCAG GTATGGTCCA TAGATGTGAC CTATTGCCGA ATGAAACGCG GTTTCATGTA TATGGTTGCA ATAATAGACT
9  GGTATTCTCG GTATATTGTT GGGTTTGAAC TATCAAACAC TCTTGATAAG ACATTCGTCA TAGAAGCAAT CCAAAAGGCC ATAAAGCGAT ATGGCAAGCC
10 TGAAATCATG AACAGTGATC AAGGCTCACA GTTTACCAGT GATGATTACA TAAATCTATT AAAAAATAAC GGTATCAAAA TATCTATGGA TGGAAAAGGA
11 AGAGCATTAG ACAACCAAAG GATAGAACGA TTTTTCCGTT CCTACAAGTG GGAGAAACTT TATCTTGAAG AGTGCGAAAC GGTACAACAA CTTAGACAAA
12 TCACAAAGGA ATATGTGGAG CACTATAACC ATAGGAGACC GCACCAGTCA TTGGATTACA AAACACCGGC AGAGTATTAC TTTGGAGGAT ATGACCAGCT
13 ACTGGCAGTT GTATAGAATT ATGGGGCTCC GCCCCAAACC CCGTCCTCAC CGGAAGGCAG CCGGTCTGTC ATAACAGACC GGAAAGCAAA AGGATATATG
14 TCCAAAGGAT GTCAAGGGTC AAGATGAACT CGCTTACGCT CGCCCTTGAC ATCCTCCAAC AGAGTGCACA GTTGTAAAGA TTATACAAAA TTAAGAAAGG
15 AGAGCTAACT TAGAAAAGCT AAAAAACTGT CTTGACAATG GGGAGCATTA TAAATCCTCC TATTTCCAAT CAAGGCTGGG AACATTTCCC TCACCGCCAA
16 ATAATCTTTT TTTCTTTCCC GTCTTATAAT CCAGAGCATA TATTATATA
```

FIG. S3. Nucleic acid sequences of the insert sequences (A) ISCth1 and (B) IS120 in *C. thermocellum*.

TABLE S1. Suppliers of chemicals and gases.

Chemical/gas	Chemostats and single-colony isolation[a]	Plate-reader cultivations, strain construction and enzyme assays[b]
Avicel PH105	FMC Biopolymer (Philadelphia, PA, USA)	
Cellobiose	Sigma-Aldrich[c]	Sigma-Aldrich[d]
Fructose	Sigma-Aldrich[c]	Sigma-Aldrich[d]
Glucose	Sigma-Aldrich[c]	Sigma-Aldrich[d]
MOPS sodium salt	Sigma-Aldrich[c]	Sigma-Aldrich[d]
Na_3-citrate dehydrate	N.A.	Merck Chemicals and Life Science[e]
Yeast extract	N.A.	VWR International[f]
D-biotin	Sigma-Aldrich[c]	Sigma-Aldrich[d]
P-aminobenzoic acid	Sigma-Aldrich[c]	Sigma-Aldrich[d]
Pyridoxamine-$(HCl)_2$	Sigma-Aldrich[c]	Sigma-Aldrich[d]
Vitamin B12	Sigma-Aldrich[c]	Sigma-Aldrich[d]
$(NH_4)_2SO_4$	N.A.	Merck Chemicals and Life Science[e]
Resazurin	N.A.	Sigma-Aldrich[d]
$CaCl_2 \cdot 2H_2O$	Sigma-Aldrich[c]	Sigma-Aldrich[d]
K_2HPO_4	Fisher Scientific[g]	Sigma-Aldrich[d]
$FeCl_2 \cdot 4H_2O$	Sigma-Aldrich[c]	Sigma-Aldrich[d]
$FeSO_4 \cdot 7H_2O$	Sigma-Aldrich[c]	ACROS Organics[h]
L-cysteine-HCl-H_2O	Sigma-Aldrich[c]	Sigma-Aldrich[d]
$MgCl_2 \cdot 6H_2O$	Sigma-Aldrich[c]	Sigma-Aldrich[d]
KH_2PO4	Fisher Scientific[g]	VWR International[f]
Na_2SO_4	Sigma-Aldrich[c]	Sigma-Aldrich[d]
KOH	Fisher Scientific[g]	Sigma-Aldrich[d]
Urea	Fisher Scientific[g]	Sigma-Aldrich[d]
5-fluoro-2'-deoxyuradine	N.A.	Sigma-Aldrich[d]
8-Azahypoxanthine	N.A.	ACROS Organics (VWR International[f])
Thiamphenicol	N.A.	Sigma-Aldrich[d]
K_3-citrate monohydrate	Sigma-Aldrich[c]	N.A.
Citric acid monohydrate	Sigma-Aldrich[c]	N.A.
$NaHCO_3$	Sigma-Aldrich[c]	N.A.
Agar, pure powder	ACROS Organics (Fisher Scientific[g])	VWR International[f]
H_3BO_3	Sigma-Aldrich[c]	Sigma-Aldrich[d]
$CoCl_2 \cdot 6H_2O$	Sigma-Aldrich[c]	Sigma-Aldrich[d]
$CuSO_4 \cdot 5H_2O$	Sigma-Aldrich[c]	Sigma-Aldrich[d]
$MnCl_2 \cdot 4H_2O$	Sigma-Aldrich[c]	ACROS Organics[h]
$NiCl_2 \cdot 6H_2O$	Sigma-Aldrich[c]	Sigma-Aldrich[d]

$Na_2MoO_4 \cdot 2H_2O$	Sigma-Aldrich[c]	ACROS Organics[h]
$ZnCl_2$	Sigma-Aldrich[c]	Sigma-Aldrich[d]
Gas: 5 % H_2, 10 % CO_2 and 85 % N_2	Airgas[i]	Strandmöllen (Ljungby, SE)
Gas: 20 % CO_2 and 80 % N_2	Airgas[i]	Strandmöllen (Ljungby, SE)
N_2 gas (N5.0)	Airgas[i]	Nippon Gases (Köping, SE)
Tris-HCl	N.A.	Merck Chemicals and Life Science[e]
$NADP^+$ disodium	N.A.	Roche[d]
NADH dipotassium	N.A.	Sigma-Aldrich[d]
KCl	N.A.	Merck Chemicals and Life Science[e]
D-Fructose 1,6-diphosphate	N.A.	Sigma-Aldrich[d]
Sodium pyruvate	N.A.	Sigma-Aldrich[d]
ATP disodium hydrate	N.A.	Sigma-Aldrich[d]
GTP sodium hydrate	N.A.	Sigma-Aldrich[d]
Bovine serum albumin	N.A.	Sigma-Aldrich[d]

[a] Performed at Dartmouth College (USA).
[b] Performed at KTH (SE).
[c] St. Louis, MO, USA
[d] Stockholm, SE
[e] Solna, SE
[f] Spånga, SE
[g] Pittsburgh, PA, USA
[h] From Fisher Scientific (Hägersten, SE)
[i] White River Junction, VT, USA
N.A. not applicable

TABLE S2. Lactate dehydrogenase activity in cell-free extracts from LL345 and AVM013 (Δ*clo1313_1831*) grown on different carbon sources.[a,b]

			**Lactate dehydrogenase activity (U $mg_{protein}^{-1}$)**	
**Strain**	**Carbon source**	**Cofactor**	in cell-free extracts used for the glucokinase assay	in cell-free extracts used for the fructokinase assay
LL345	cellobiose	ATP	0.65 ± 0.04	0.65 ± 0.01
AVM013	cellobiose	ATP	0.58 ± 0.03	0.44 ± 0.02
AVM013	glucose	ATP	0.63 ± 0.03	0.87 ± 0.06
AVM013	fructose	ATP	0.96 ± 0.06	0.82 ± 0.02

[a] Activities are reported as units (U) per mg of total protein in the cell-free extract, where one U is equal to converting one µmol of substrate in one minute.
[b] Data are shown as average ± standard deviation (*n* = 4).

TABLE S3. Measurement data from cellobiose-, glucose- or fructose-limited chemostats at a dilution rate of 0.10 h^{-1} with *C. thermocellum*.[a]

	Cellobiose		Glucose		Fructose	
**Physiological parameter**	**ave**	**SD**	**ave**	**SD**	**ave**	**SD**
Optical density	1.41	0.07	1.01	0.07	1.25	0.08
CDW (g L^{-1})	0.71	0.05	0.45	0.02	0.79	0.01
TOC (gC L^{-1})	0.33	0.01	0.22	0.02	0.34	0.00
TN (gN L^{-1})	0.09	0.00	0.06	0.01	0.09	0.00
Cellobiose, feed (g L^{-1})	4.70	0.01	<0.01		<0.01	
Glucose, feed (g L^{-1})	<0.01		4.77	N.A.	<0.01	
Fructose, feed (g L^{-1})	<0.01		<0.01		4.83	N.A.
Cellobiose, residual (g L^{-1})	< 0.01		< 0.01		< 0.01	
Glucose, residual (g L^{-1})	0.02	0.00	0.02	0.00	0.01	0.00
Fructose, residual (g L^{-1})	< 0.01		< 0.01		0.51	0.02
Acetate (g L^{-1})	0.95	0.02	1.34	0.05	0.66	0.08
Ethanol (g L^{-1})	0.82	0.02	0.64	0.06	0.51	0.00
Formate (g L^{-1})	0.22	0.03	0.28	0.02	0.17	0.00
Lactate (g L^{-1})	0.04	0.01	0.14	0.04	0.01	0.01
Pyruvate (g L^{-1})	0.02	0.00	0.11	0.00	0.04	0.02
Malate (g L^{-1})	0.01	0.01	0.03	0.04	< 0.01	

[a] Data are shown for four biological replicates on cellobiose and two biological replicates on glucose and fructose.

N.A. not applicable since the duplicate chemostats shared the same feed vessel.

TABLE S4. Biomass and product yields on consumed substrate normalized to grams of carbon (gC) from cellobiose-, glucose- or fructose-limited chemostats at a dilution rate of 0.10 h^{-1}.[a]

	**Cellobiose**		**Glucose**		**Fructose**	
**Yield (gC gC^{-1})**	**Average**	**SD**	**Average**	**SD**	**Average**	**SD**
Biomass[b]	16.8	0.5	11.7	1.3	19.7	0.0
Acetate	19.3	0.5	28.7	1.0	15.4	1.8
Ethanol	21.4	0.5	17.6	1.7	15.2	0.1
Formate	2.9	0.4	4.0	0.2	2.6	0.0
Lactate	0.7	0.1	2.9	0.8	0.2	0.3
Pyruvate	0.4	0.1	2.4	0.1	0.9	0.5
Malate	0.2	0.2	0.5	0.7	0.0	0.0
CO_2[c]	17.4	0.7	19.2	0.1	12.8	1.0
Carbon recovery (%)[d]	79.2	1.2	87.0	1.2	66.8	3.7

[a] Data are shown as average and standard deviation (SD) of four biological replicates on cellobiose and two biological replicates on glucose and fructose.

[b] Based on TOC.

[c] The CO_2 yield was calculated as the sum of the molar yields of ethanol and acetate subtracted with formate.

[d] Calculated as the ratio between total carbon in products and total carbon in consumed substrate.

TABLE S5. Specific growth rate and lag time on 5 g L^{-1} cellobiose, glucose, and fructose, from a plate reader experiment with wild type and evolved strains from glucose-limited (G-LL1516 – G-LL1523) and fructose-limited (F-LL1538 – F-LL1545) chemostats. The inocula for the plate reader were grown on cellobiose.[a]

	Growth rate (h^{-1})			Lag time (h)		
**Strain**	**Cellobiose**	**Glucose**	**Fructose**	**Cellobiose**	**Glucose**	**Fructose**
Wild type	0.50 ± 0.07	0.25 ± 0.04	N.D.	0 ± 0	42 ± 4	N.D.
G-LL1516	0.34 ± 0.01	0.23 ± 0.01	N.D.	1.1 ± 0.5	2.4 ± 0.7	N.D.
G-LL1517	N.A.	N.A.	N.A.	0 ± 0	0 ± 0	N.A.
G-LL1518	0.45 ± 0.03	0.18 ± 0.01	N.D.	0 ± 0	13 ± 0.4	N.D.
G-LL1519	0.38 ± 0.01	0.31 ± 0.00	N.D.	0 ± 0	3.1 ± 0.2	N.D.
G-LL1520	0.20 ± 0.01	0.24 ± 0.02	0.17 ± 0.02	0 ± 0	1.9 ± 0.2	42 ± 7
G-LL1521	0.30 ± 0.02	0.28 ± 0.02	N.D.	10 ± 1	1.8 ± 0.4	N.D.
G-LL1522	0.33 ± 0.02	0.18 ± 0.01	N.D.	0 ± 0	10 ± 1	N.D.
G-LL1523	0.37 ± 0.02	0.30 ± 0.02	N.D.	0.4 ± 0.6	2.3 ± 0.1	N.D.
F-LL1538	0.41 ± 0.03	0.25 ± 0.01	0.44 ± 0.05	0 ± 0	3 ± 0.3	0 ± 0
F-LL1539	0.32 ± 0.02	0.22 ± 0.01	0.35 ± 0.02	0 ± 0	2.6 ± 0.3	0 ± 0
F-LL1540	0.29 ± 0.02	0.20 ± 0.02	0.33 ± 0.02	1.9 ± 0.6	3.6 ± 0.2	1.1 ± 0.2
F-LL1541	0.33 ± 0.02	0.23 ± 0.01	0.36 ± 0.02	0 ± 0	2.8 ± 0.2	0 ± 0
F-LL1542	0.39 ± 0.04	0.19 ± 0.01	0.39 ± 0.03	0 ± 0	2.9 ± 0.4	0 ± 0
F-LL1543	0.42 ± 0.05	0.17 ± 0.01	0.47 ± 0.03	0 ± 0	1.5 ± 0.7	0 ± 0
F-LL1544	0.39 ± 0.04	0.21 ± 0.00	0.34 ± 0.01	3.4 ± 2.2	1.8 ± 0.3	0.7 ± 0.5
F-LL1545	0.47 ± 0.02	0.31 ± 0.03	0.47 ± 0.03	1.5 ± 1.8	1.1 ± 0.4	0 ± 0

[a] Data are presented as average and standard deviation (SD) for $n \geq 3$ replicates. For wild type, $n = 24$. N.D. (not detected) represents no detected growth within the time-frame of the cultivation (80 hours). N.A. (not applicable) represents experimental error in obtaining growth on the plate reader.

TABLE S6. Specific growth rate and lag time on cellobiose, glucose, and fructose, from a plate reader experiment with evolved strains from glucose-limited (G-LL1516 – G-LL1523) and fructose-limited (F-LL1538 – F-LL1545) chemostats. Each inoculum for the plate reader was grown on the hexose sugar the respective strain was isolate in.[a]

	**Growth rate (h^{-1})**			**Lag time (h)**		
**Strain**	**Cellobiose**	**Glucose**	**Fructose**	**Cellobiose**	**Glucose**	**Fructose**
G-LL1516	0.37 ± 0.01	0.30 ± 0.01	N.D.	1.9 ± 0.5	2.2 ± 1.4	N.D.
G-LL1517	0.22 ± 0.03	0.27 ± 0.03	0.05 ± 0.01	12 ± 1	8.5 ± 1.5	9.0 ± 0.5
G-LL1518	0.25 ± 0.03	0.37 ± 0.01	N.D.	29 ± 2	1.7 ± 0.6	N.D.
G-LL1519	0.50 ± 0.04	0.22 ± 0.02	0.05 ± 0.01	15 ± 1	7.0 ± 3.1	21 ± 11
G-LL1520	0.31 ± 0.03	0.23 ± 0.01	0.18 ± 0.02	26 ± 1	1.1 ± 1.5	30 ± 1
G-LL1521	N.D.	0.28 ± 0.02	N.D.	N.D.	4.5 ± 0.7	N.D.
G-LL1522	0.34 ± 0.06	0.30 ± 0.01	N.D.	22 ± 1	0 ± 0	N.D.
G-LL1523	0.36 ± 0.04	0.39 ± 0.01	N.D.	3.0 ± 0.4	0 ± 0	N.D.
F-LL1538	0.51 ± 0.04	0.30 ± 0.06	0.49 ± 0.06	0 ± 0	1.6 ± 0.5	0 ± 0
F-LL1539	0.35 ± 0.01	0.24 ± 0.02	0.39 ± 0.04	0.1 ± 0.3	7.9 ± 0.7	1.6 ± 1.2
F-LL1540	0.35 ± 0.01	0.22 ± 0.02	0.36 ± 0.04	0 ± 0	1.3 ± 0.3	3.7 ± 0.8
F-LL1541	0.43 ± 0.04	0.20 ± 0.01	0.42 ± 0.04	0 ± 0	2.1 ± 0.3	0.7 ± 0.9
F-LL1542	0.41 ± 0.03	0.22 ± 0.02	0.46 ± 0.03	1.8 ± 1.7	3.8 ± 0.9	1.6 ± 0.5
F-LL1543	0.39 ± 0.03	0.19 ± 0.02	0.41 ± 0.04	3.6 ± 1.3	8.7 ± 4.1	0 ± 0
F-LL1544	0.37 ± 0.03	0.16 ± 0.00	0.40 ± 0.02	3.6 ± 1.6	8.3 ± 1.7	0 ± 0
F-LL1545	0.38 ± 0.03	0.16 ± 0.01	0.31 ± 0.02	3.1 ± 1.8	3.6 ± 0.8	0.2 ± 0.3

[a] Data are presented as average and standard deviation (SD) for ≥ 3 replicates. N.D. represents no detected growth within the time-frame of the cultivation (80 hours).
